# Supplementary material for: Lamin A/C-dependent chromatin architecture safeguards naïve pluripotency to prevent aberrant cardiovascular cell fate and function
Source: Nat Commun. 2022 Nov 4;13:6663. doi: 10.1038/s41467-022-34366-7 (PMC9636150; doi:10.1038/s41467-022-34366-7)

Supplementary Data for

Lamin A-dependent chromatin architecture safeguards naïve pluripotency to prevent aberrant cardiovascular cell fate and function

Yinuo Wang[1,2], Adel Elsherbiny[1], Linda Kessler[1], Julio Cordero[1,2], Haojie Shi[1,2], Heike Serke[1,2], Olga Lityagina[1], Felix A. Trogisch[2,3], Mona Malek Mohammadi[2,3,7], Ibrahim El-Battrawy[4,8], Johannes Backs[2,5], Thomas Wieland[2,6], Joerg Heineke[2,3] and Gergana Dobreva[1,2*]

*Corresponding author, email: Gergana.Dobreva@medma.uni-heidelberg.de

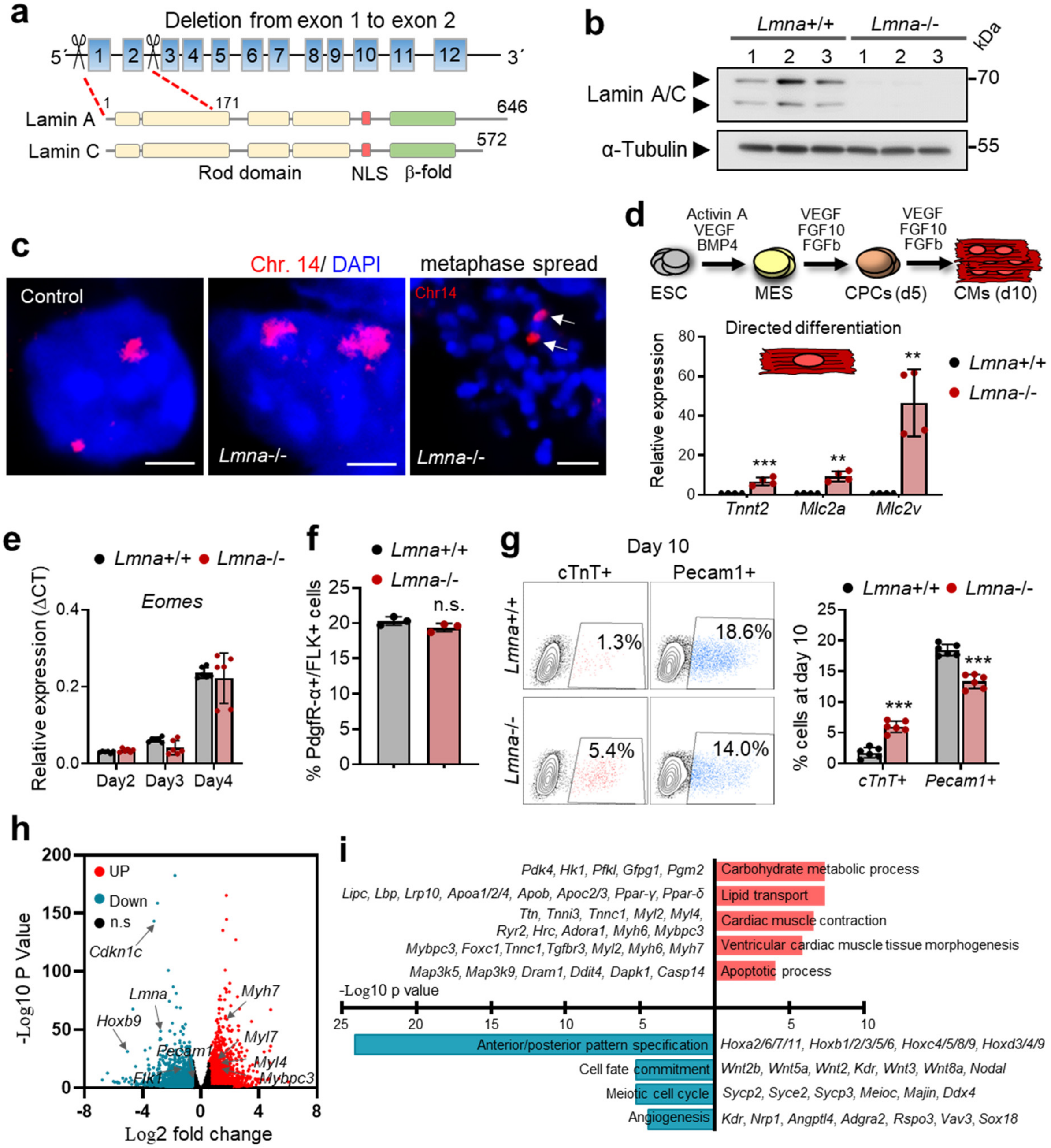

Supplementary Fig.1: Role of lamin A/C in cardiovascular differentiation.

a Schematic diagram of *Lmna* knockout ESC generation by CRISPR/Cas9 gene editing. **b** Western blot analysis of lamin A/C in control and *Lmna*-/- ESCs. **c** FISH chromosome painting of Chr. 14 in control and *Lmna*-/- mESCs, showing large-scale chromatin decondensation of Chr. 14 in the interphase nucleus and expanded chromosome territory in *Lmna*-/- ESCs (middle

panel) compared to control cells (left panel), while the chromosome number was not changed as assessed by metaphase spread with whole-chromosome paint probe for Chr. 14 in red. Scale bars, 4 μm. **d** Schematic diagram of directed differentiation of ESCs into CMs (up). Relative mRNA expression of CM genes at day 10 of directed cardiac differentiation (down, n= 4). **e** Relative mRNA expression of the mesodermal marker *Eomes* in EBs differentiated from control and *Lmna*-/- mESCs at different days (n=6). **f** Percentage of early PdgfR-α+/FLK+ cardiovascular precursors in d4 EBs differentiated from control and *Lmna*-/- ESCs determined by FACS analysis (n=3). **g** Representative FACS analyses of cTnT+ CMs and Pecam1+ ECs (left) and percentage of cTnT+ CMs and Pecam1+ ECs determined by FACS (right) at day 10 of ESC differentiation (n=6). **h** Volcano plots showing the distribution of differentially expressed genes in *Lmna*+/+ and *Lmna*-/- day 10 EBs. n=3 biologically independent samples; $\log 2$ fold change $\leqslant$ -0.58, $\geqslant$0.58; p-value < 0.05. P-value was determined by DESeq2 algorithm. **i** Heatmap and Gene ontology (GO) analysis of up-regulated (red) and down-regulated (blue) genes in d10 EBs. Significance is presented as - $\log 10$ enrichment p-values from pathway analysis using DAVID Bioinformatics Resources 6.8. **d**, **e**, **f**, **g** Data are presented as mean ± SD and p-values were determined by unpaired two-tailed Student's t-test. P-values are as follows: ***$p < 0.001$, **$p < 0.01$, *$p < 0.05$. Source data are provided as a Source Data file.

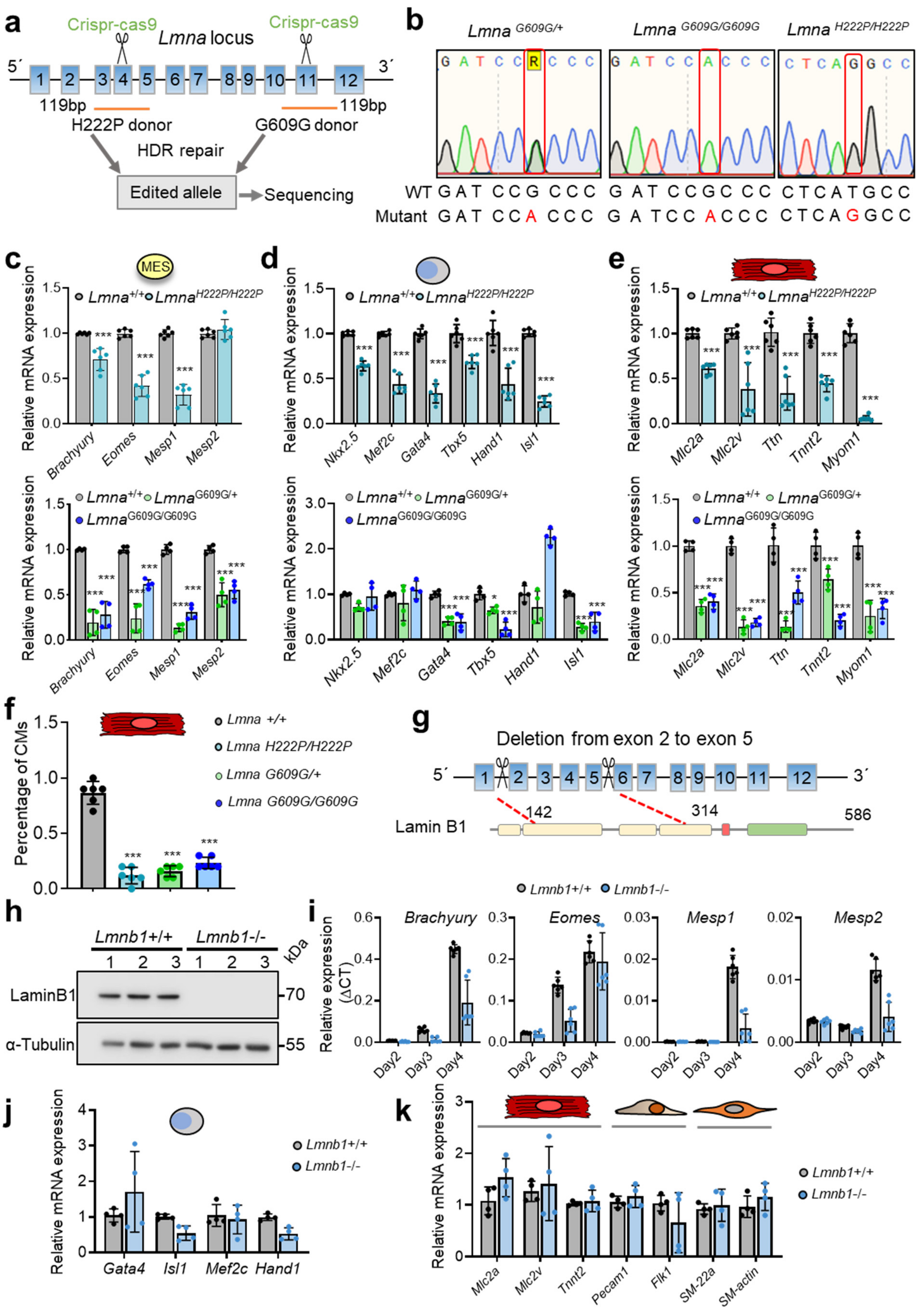
a
Crispr-cas9
Lmna locus
Crispr-cas9
5´
1 2 3 4 5 6 7 8 9 10 11 12
3´
119bp
119bp
H222P donor
G609G donor
HDR repair
Edited allele
Sequencing
b
Lmna G609G/+
Lmna G609G/G609G
Lmna H222P/H222P
WT GAT CCG CCC GATCCGCCC CTCATGCC
Mutant GAT CCA CCC GATCCACCC CTCAGGCC
c
MES
Lmna+/+ LmnaH222P/H222P
Relative mRNA expression
Brachyury Eomes Mesp1 Mesp2
Lmna+/+ LmnaG609G/+ LmnaG609G/G609G
d
Nkx2.5 Mef2c Gata4 Tbx5 Hand1 Isl1
e
Mlc2a Mlc2v Ttn Tnnt2 Myom1
f
Percentage of CMs
Lmna +/+
Lmna H222P/H222P
Lmna G609G/+
Lmna G609G/G609G
g
Deletion from exon 2 to exon 5
Lamin B1
142
314
586
h
Lmnb1+/+
Lmnb1-/-
kDa
LaminB1
α-Tubulin
70
55
i
Lmnb1+/+ Lmnb1-/-
Relative expression (ΔCT)
Brachyury Eomes Mesp1 Mesp2
Day2 Day3 Day4
j
Relative mRNA expression
Gata4 Isl1 Mef2c Hand1
Lmnb1+/+
Lmnb1-/-
k
Relative mRNA expression
Mlc2a Mlc2v Tnnt2 Pecam1 Flk1 SM-22a SM-actin
Lmnb1+/+
Lmnb1-/-

Supplementary Fig. 2: Impact of lamin A/C mutations and lamin B1 on cardiomyocyte differentiation.

a Schematic diagram of the strategy used for *Lmna* pH222P and G609G mutation generation. **b** Representative Sanger sequencing traces confirming the successful mutation of *Lmna* in ESCs. **c** Relative mRNA expression of the mesodermal markers in EBs differentiated from control and *Lmna H222P/H222P* (top) (n=6) as well as control, *Lmna G609G/+* and *Lmna G609G/G609G* mESCs (bottom) (n=4) at day 4. **d** Relative mRNA expression of cardiac progenitor markers in EBs differentiated from control and *Lmna H222P/H222P* (top) (n=6) as well as control, *Lmna G609G/+* and *Lmna G609G/G609G* mESCs (bottom) (n=4) at day 6. **e** Relative mRNA expression of the cardiomyocyte marker genes in EBs differentiated from control and *Lmna H222P/H222P* (top) (n=6) as well as control, *Lmna G609G/+* and *Lmna G609G/G609G* mESCs (bottom) (n=4) at day 8. **f** Percentage of cTnT+ CMs determined by FACS at day 8 of ESC differentiation (n=6). Data are presented as mean ± SD; One-way ANOVA with Tukey correction comparisons were used; P-values are as follows: ***$p < 0.001$, **$p < 0.01$, *$p < 0.05$. **g** Schematic representation of the strategy used to generate *Lmnb1*-/- ESCs using CRISPR/Cas9 gene editing. **h** Western blot analysis of lamin B1 in control and *Lmnb1*-/- ESC. **i** Relative mRNA expression of mesodermal markers in EBs at different days (n=6). **j** Relative mRNA expression of cardiac progenitor marker genes in d6 EBs (n=4). **k** Relative mRNA expression of CM (*Mlc2a*, *Mlc2v* and *Tnnt2*), EC (*Flk1* and *Pecam1*), and smooth muscle (*SM-22a*, *SM-actin*) genes in d10 EBs (n=4). **c-e**: Data are presented as mean ± SD, unpaired two-tailed Student's t-test (top panel) and One-way ANOVA with Tukey correction comparisons (bottom panel) were used. P-values are as follows: ***$p < 0.001$, **$p < 0.01$, *$p < 0.05$. **i-k**: Data are presented as mean ± SD, unpaired two-tailed Student's t-test (top panel) were used. P-values are as follows: ***$p < 0.001$, **$p < 0.01$, *$p < 0.05$. Source data are provided as a Source Data file.

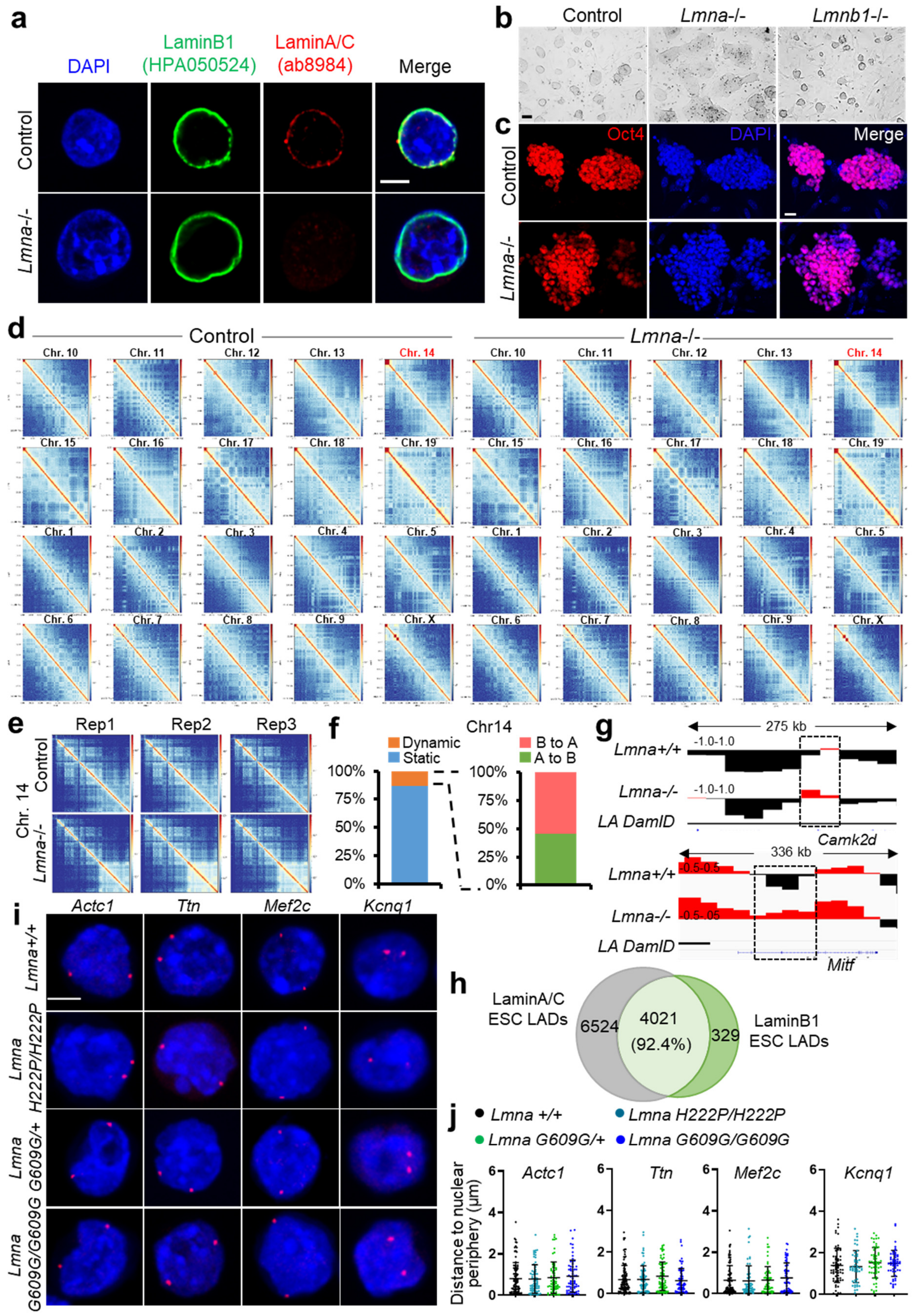

a
DAPI
LaminB1 (HPA050524)
LaminA/C (ab8984)
Merge
Control
Lmna-/-
b
Control
Lmna-/-
Lmnb1-/-
c
Oct4
DAPI
Merge
Control
Lmna-/-
d
Control
Lmna-/-
Chr. 10
Chr. 11
Chr. 12
Chr. 13
Chr. 14
Chr. 15
Chr. 16
Chr. 17
Chr. 18
Chr. 19
Chr. 1
Chr. 2
Chr. 3
Chr. 4
Chr. 5
Chr. 6
Chr. 7
Chr. 8
Chr. 9
Chr. X
e
Rep1
Rep2
Rep3
Chr. 14
Control
Lmna-/-
f
Chr14
Dynamic
Static
B to A
A to B
100%
75%
50%
25%
0%
g
275 kb
Lmna+/+
Lmna-/-
LA DamID
Camk2d
336 kb
Mitf
h
LaminA/C ESC LADs
LaminB1 ESC LADs
6524
4021 (92.4%)
329
i
Actc1
Ttn
Mef2c
Kcnq1
Lmna+/+
Lmna H222P/H222P
Lmna G609G/+
Lmna G609G/G609G
j
Lmna +/+
Lmna H222P/H222P
Lmna G609G/+
Lmna G609G/G609G
Distance to nuclear periphery (µm)

Supplementary Fig. 3: Nuclear and 3D genome architecture alterations in *Lmna*-/- ESCs.

a Confocal images of immunostaining for lamin A/C (red), lamin B1 (green) and nucleus (DAPI, blue) in *Lmna*+/+ and *Lmna*-/- ESCs. Scale bars, 4 µm. **b** Phase contrast images showing colony morphology of control, *Lmna*-/- and *Lmnb1*-/- ESC colonies. Scale bars, 120 µm. **c** Confocal images of immunostaining for Oct4 (red) and nucleus (DAPI, blue) in *Lmna*+/+ and *Lmna*-/-ESCs. Scale bars, 20 µm. **d** Log-transformed contact matrices of all chromosomes at 100 kb resolution in *Lmna*+/+ and *Lmna*-/- ESCs. **e** Log-transformed contact matrices of Chr. 14 at 100 kb resolution in *Lmna*+/+ and *Lmna*-/- biological triplicates. **f** Static (blue) and dynamic (orange) compartments at Chr. 14. Regions with A/B compartment switch are subdivided into A to B (green) and B to A (pink) transitions. **g** Genome tracks of Hi-C PC1 at 25 kb resolution and lamin A/C DamID regions at cardiac genes transiting from B (black) compartment to A (red) compartment in *Lmna*-/- ESCs. **h** Overlap of genes within lamin A/C and lamin B1 LADs. **i** Representative DNA FISH images of cardiac-specific genes found within LADs showing no re-localization from the nuclear periphery to the nuclear interior in *Lmna H222P/H222P* as well as *Lmna G609G/+* and *Lmna G609G/G609G* mESCs. *Kcnq1*, a gene not found in LADs, also did not show any change in localization. Scale bars, 4 µm. **j** Quantification of the distance of *Actc1*, *Ttn, Mef2c* and *Kcnq1* to the nuclear periphery in individual nuclei of *Lmna+/+* and *Lmna H222P/H222P*, *Lmna G609G/+* and *Lmna G609G/G609G* mESCs. 30-50 cells were quantified from each group. Data are presented as mean ± SD; One-way ANOVA with Tukey correction comparisons were used; P-values are as follows: ***$p < 0.001$, **$p < 0.01$, *$p < 0.05$. Source data are provided as a Source Data file.

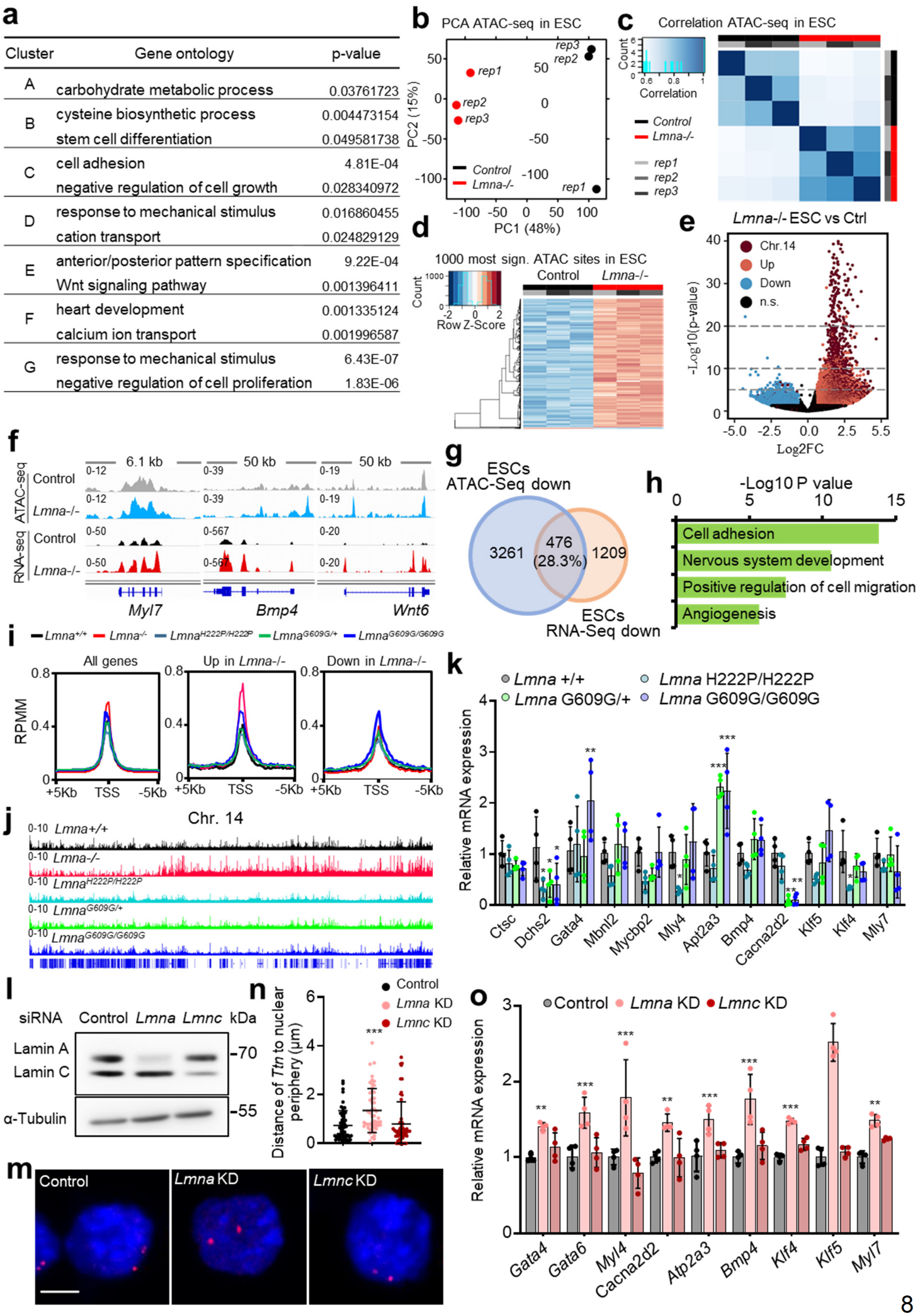
a
Cluster
Gene ontology
p-value
A carbohydrate metabolic process 0.03761723
B cysteine biosynthetic process 0.004473154
stem cell differentiation 0.049581738
C cell adhesion 4.81E-04
negative regulation of cell growth 0.028340972
D response to mechanical stimulus 0.016860455
cation transport 0.024829129
E anterior/posterior pattern specification 9.22E-04
Wnt signaling pathway 0.001396411
F heart development 0.001335124
calcium ion transport 0.001996587
G response to mechanical stimulus 6.43E-07
negative regulation of cell proliferation 1.83E-06
b
PCA ATAC-seq in ESC
PC2 (15%)
PC1 (48%)
Control
Lmna-/-
c
Correlation ATAC-seq in ESC
Correlation
Control
Lmna-/-
rep1
rep2
rep3
d
1000 most sign. ATAC sites in ESC
Row Z-Score
Control
Lmna-/-
e
Lmna-/- ESC vs Ctrl
Chr.14
Up
Down
n.s.
-Log10(p-value)
Log2FC
f
ATAC-seq
RNA-seq
Control
Lmna-/-
Myl7
Bmp4
Wnt6
g
ESCs
ATAC-Seq down
3261
476
(28.3%)
1209
ESCs
RNA-Seq down
h
-Log10 P value
Cell adhesion
Nervous system development
Positive regulation of cell migration
Angiogenesis
i
All genes
Up in Lmna-/-
Down in Lmna-/-
RPMM
TSS
j
Chr. 14
k
Lmna +/+
Lmna H222P/H222P
Lmna G609G/+
Lmna G609G/G609G
Relative mRNA expression
Ctsc
Dchs2
Gata4
Mbnl2
Mycbp2
Mly4
Atp2a3
Bmp4
Cacna2d2
Klf5
Klf4
Mly7
l
siRNA
Control
Lmna
Lmnc
kDa
Lamin A
Lamin C
α-Tubulin
m
Control
Lmna KD
Lmnc KD
n
Distance of Ttn to nuclear periphery (µm)
o
Control
Lmna KD
Lmnc KD
Relative mRNA expression
Gata4
Gata6
Myl4
Cacna2d2
Atp2a3
Bmp4
Klf4
Klf5
Myl7

Supplementary Fig. 4: Transcriptional and chromatin alterations in *Lmna*-/- and *Lmna*-mutant ESCs.

a Representative GO terms in the different clusters presented in Fig. 3e. **b** Principal component analysis (PCA) of genome-wide chromatin accessibility variation in *Lmna*+/+ and *Lmna*-/- ESCs. n=3 biologically independent samples**. c** Correlation heatmap of RPKM normalized ATAC-seq reads in *Lmna*+/+ and *Lmna*-/- ESCs. **d** Heatmap of 10000 most significantly altered ATAC-Seq peaks between *Lmna*+/+ and *Lmna*-/- CMs. **e** Volcano plot showing the distribution of differentially accessible chromatin regions between *Lmna*+/+ and *Lmna*-/- ESCs. Most significantly altered chromatin regions are located on Chr. 14. n=3 biologically independent samples; Log2 fold change ≤ -0.58, ≥0.58; p-value < 0.05. P-value was determined by DESeq2 algorithm. **f** Examples of genes showing increased chromatin accessibility and transcriptional activation in mESC. Genome tracks of ATAC-Seq and RNA-Seq reads of *Lmna*+/+ and *Lmna*-/- mESC are presented. **g** Overlap of genes showing decreased ATAC-Seq signal and decreased expression in *Lmna*-/- mESC. **h** GO terms of genes within the overlap shown in **g**. Significance is presented as - Log10 enrichment p-values from pathway analysis using DAVID Bioinformatics Resources 6.8. **i** Normalized ATAC-Seq signal intensity at the TSS ± 5 kb of all genes (left) as well as genes upregulated (middle) and downregulated (right) upon lamin A/C loss of function in *Lmna+/+, Lmna*-/-, *Lmna H222P/H222P*, *Lmna G609G/*+ and *Lmna G609G/G609G* ESCs**. j** Genome tracks of ATAC-Seq reads in *Lmna*+/+, *Lmna*-/-, *Lmna H222P/H222P*, *Lmna G609G/*+ and *Lmna G609G/G609G* ESCs at Chr. 14. **k** Relative mRNA expression of genes upregulated upon lamin A/C loss of function in *Lmna*+/+, *Lmna H222P/H222P*, *Lmna G609G/*+ and *Lmna G609G/G609G* ESCs (n=4). **l** Western blot analysis of cells transfected with control siRNA, or siRNAs specifically targeting lamin A or lamin C. **m** Representative DNA FISH images of *Ttn* locus showing relocalization from the nuclear periphery into the nuclear interior upon lamin A but not upon lamin C depletion. Scale bars, 4 µm. **n** Quantification of the distance of *Ttn* to

nuclear periphery in individual nuclei of control, lamin A depleted or lamin C depleted mESCs (n=30-50). **o** Relative mRNA expression of genes upregulated upon lamin A/C loss of function in control, lamin A depleted or lamin C depleted mESCs (n=4). **k**, **n**, **o** Data are represented as mean ± SD; One-way ANOVA with Tukey correction comparisons was used; P-values are as follows: ***$p < 0.001$, **$p < 0.01$, *$p < 0.05$. Source data are provided as a Source Data file.

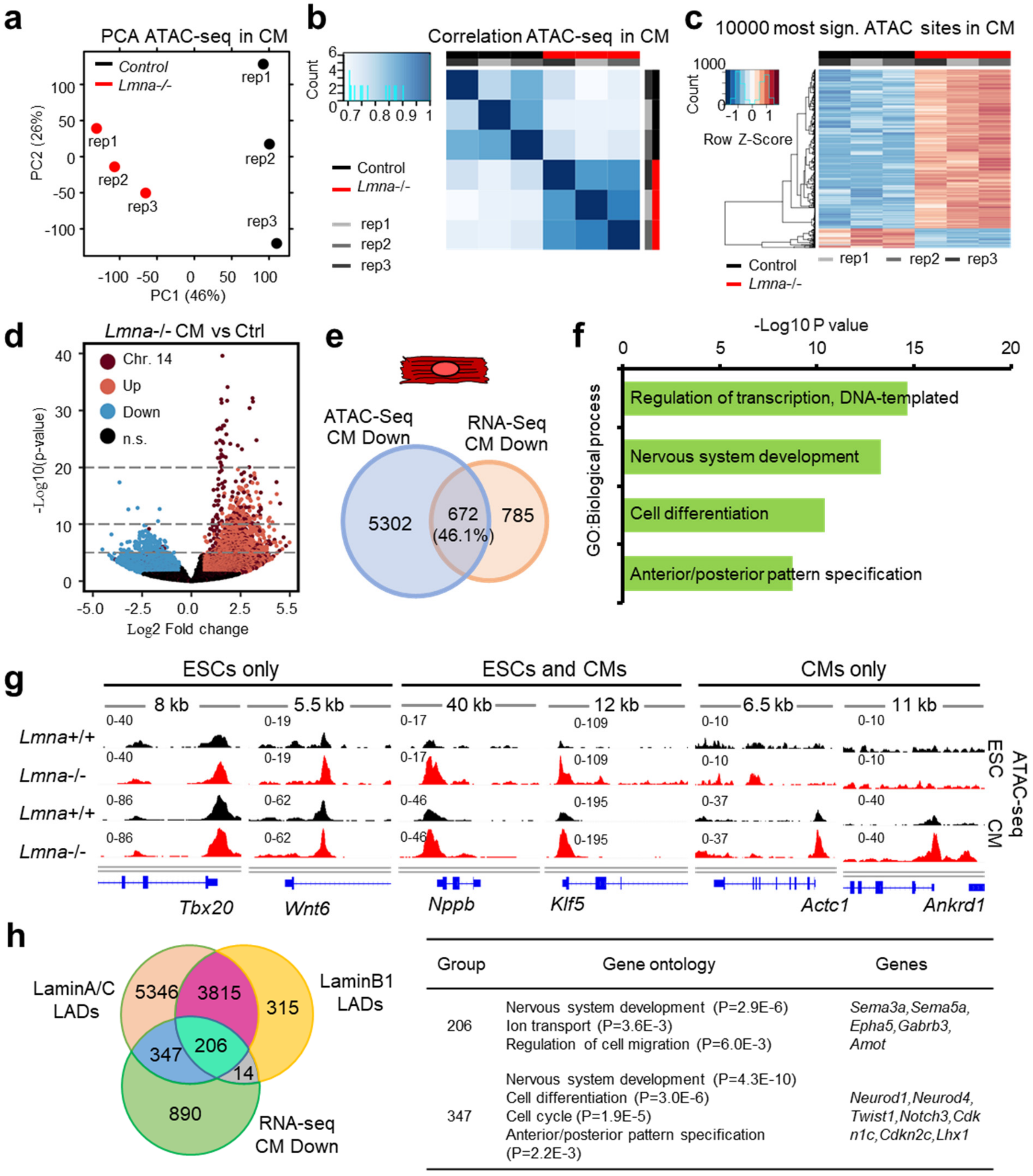

Supplementary Fig. 5: Transcriptional and chromatin alterations in *Lmna*-/- CMs.

a Principal component analysis (PCA) of genome-wide chromatin accessibility variation in *Lmna*+/+ and *Lmna*-/- CMs. **b** Correlation heatmap of ATAC-Seq reads in *Lmna*+/+ and *Lmna*-/- CMs. **c** Heatmap of 10000 most significantly altered ATAC-Seq peaks between *Lmna*+/+ and

Lmna-/- CMs. **d** Volcano plot showing the distribution of differentially accessible chromatin regions between *Lmna*+/+ and *Lmna*-/- CMs. Most significantly altered chromatin regions are located on Chr. 14. **e** Overlap of genes showing decreased ATAC-Seq signal and decreased expression in *Lmna*-/- CMs. **f** GO terms of genes within the overlap shown in **e**. Significance is presented as - Log10 enrichment p-values from pathway analysis using DAVID Bioinformatics Resources 6.8. **g** Examples of genes showing increased chromatin accessibility in *Lmna*-/- ESCs and CMs as well as genes showing increased chromatin accessibility only in ESCs or CMs. Genome tracks of ATAC-Seq reads of *Lmna*+/+ and *Lmna*-/- ESCs and CMs are presented. **h** Overlap between genes within lamin A/C and lamin B1 LADs and genes showing decreased gene expression in *Lmna*-/- CMs (left). Enriched GO terms for the genes within the different overlaps are presented in the table on the right. P-values are from pathway analysis using DAVID Bioinformatics Resources 6.8.

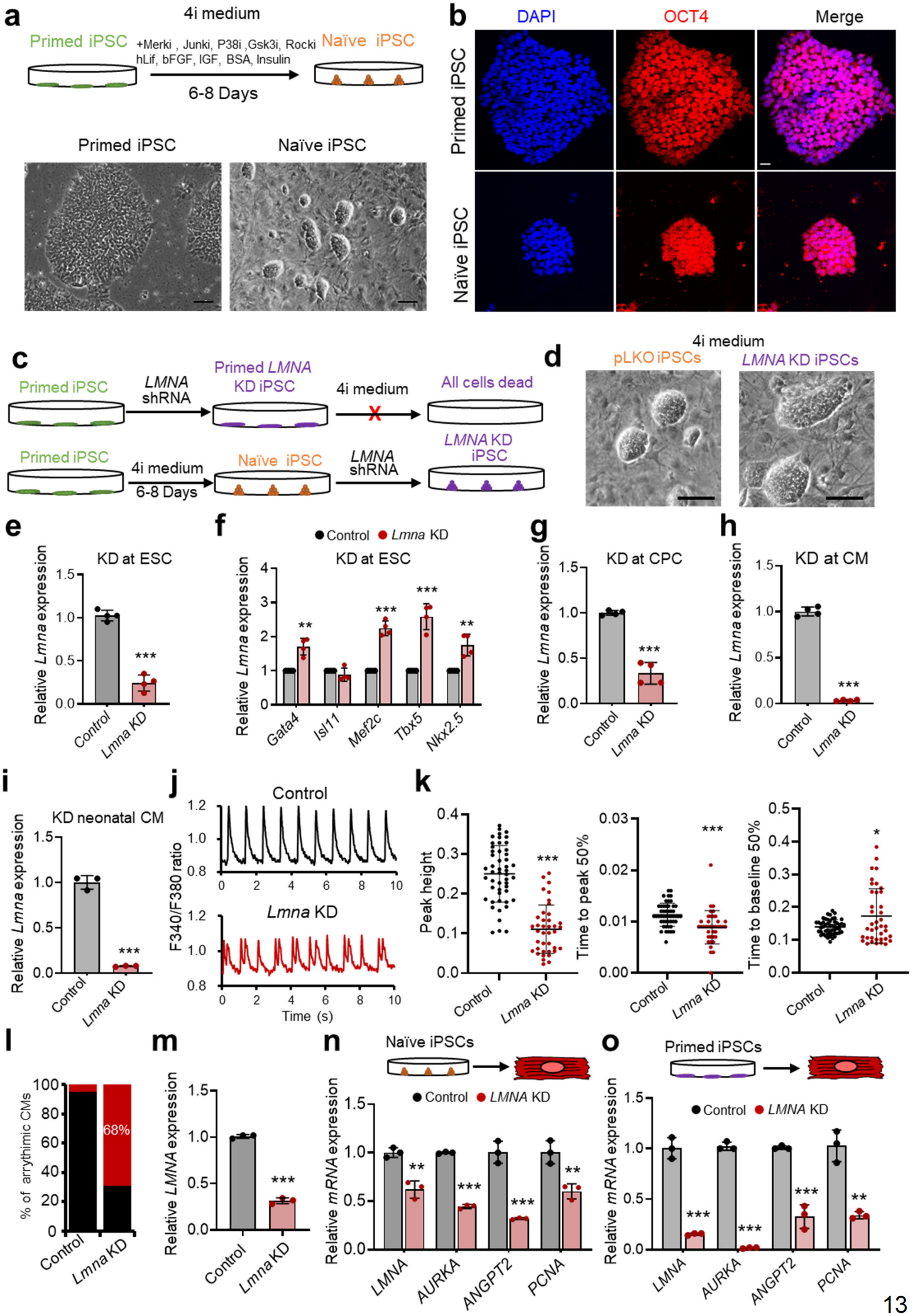

a
4i medium
Primed iPSC
+Merki , Junki, P38i ,Gsk3i, Rocki
hLif, bFGF, IGF, BSA, Insulin
Naïve iPSC
6-8 Days
Primed iPSC
Naïve iPSC
b
DAPI
OCT4
Merge
Primed iPSC
Naïve iPSC
c
Primed iPSC
LMNA shRNA
Primed LMNA KD iPSC
4i medium
All cells dead
Primed iPSC
4i medium
6-8 Days
Naïve iPSC
LMNA shRNA
LMNA KD iPSC
d
4i medium
pLKO iPSCs
LMNA KD iPSCs
e
KD at ESC
Relative Lmna expression
Control
Lmna KD
f
Control
Lmna KD
KD at ESC
Relative Lmna expression
Gata4
Isl11
Mef2c
Tbx5
Nkx2.5
g
KD at CPC
Relative Lmna expression
Control
Lmna KD
h
KD at CM
Relative Lmna expression
Control
Lmna KD
i
KD neonatal CM
Relative Lmna expression
Control
Lmna KD
j
Control
Lmna KD
F340/F380 ratio
Time (s)
k
Peak height
Time to peak 50%
Time to baseline 50%
Control
Lmna KD
l
% of arrythimic CMs
68%
Control
Lmna KD
m
Relative LMNA expression
Control
Lmna KD
n
Naïve iPSCs
Control
LMNA KD
Relative mRNA expression
LMNA
AURKA
ANGPT2
PCNA
o
Primed iPSCs
Control
LMNA KD
Relative mRNA expression
LMNA
AURKA
ANGPT2
PCNA

Supplementary Fig. 6: Lamin A/C function in naïve pluripotent stem cells.

a Schematic representation of the strategy used for conversion of primed human iPSCs into naïve hiPSCs (top panel) and representative phase contrast images showing colony morphology of hiPSC colonies cultured in standard conditions (primed state) or cultured in 4i medium (naïve state). Scale bars, 200µm. **b** Confocal images of immunostaining for OCT4 (red) and nucleus (DAPI, blue) in hiPSCs cultured in standard conditions or cultured in 4i medium on feeders. Scale bars, 20µm. **c** Schematic representation of the two different strategies used to generate *LMNA*-deficient naïve hiPSCs. **d** Representative phase contrast images showing colony morphology of naïve human iPSC after *LMNA* knockdown. Scale bars, 200µm. **e** Relative *Lmna* mRNA expression in mESC after lentiviral mediated knockdown of *Lmna* using shRNA (n=4). **f** Relative mRNA expression of cardiac progenitor marker genes at day 5 of directed cardiac differentiation after knockdown of *Lmna* by shRNA at the ESC stage (n=4). **g** Relative *Lmna* mRNA expression at d10 of directed cardiac differentiation after knockdown of *Lmna* by shRNA at the CP stage (n=4). **h** Relative *Lmna* mRNA expression at day 10 during directed cardiac differentiation after knockdown of *Lmna* by shRNA at the CM stage (day 8, n=4). **i** Relative *Lmna* mRNA expression after knockdown of *Lmna* by shRNA in isolated neonatal cardiomyocytes (n=3). **j** Representative Ca^{2+} transients traces of neonatal CMs after *Lmna* knockdown. The ratio of the fura-2 AM signal excited at 340 nm and 380 nm (F340/F380) is presented. All traces were recorded for 20 s. **k** Peak height, time to peak 50 % and time to baseline 50 % of Ca^{2+} traces of isolated neonatal cardiomyocytes after *Lmna* knockdown by shRNA. **l** Quantification of the percentage of CMs exhibiting proarrhythmic Ca^{2+} waves (red). 40-50 cells were quantified for each group. **m** Relative *LMNA* mRNA expression in naïve hiPSCs after lentiviral mediated knockdown of *LMNA* using shRNA (n=3). **n**, **o** Relative mRNA expression of genes downregulated in both DCM patients with pathogenic *LMNA* mutations and *Lmna*-/- mESCs-derived CM in human iPSCs differentiated in CMs after *LMNA* silencing in naïve (**n,** n=3) or primed hiPSCs (**o**, n=3). **e-i**, **k**, **m-o** Data are presented as mean ± SD and p

values were determined by unpaired two-tailed Student's t-test. P-values are as follows: ***$p < 0.001$, **$p < 0.01$, *$p < 0.05$. Source data are provided as a Source Data file.

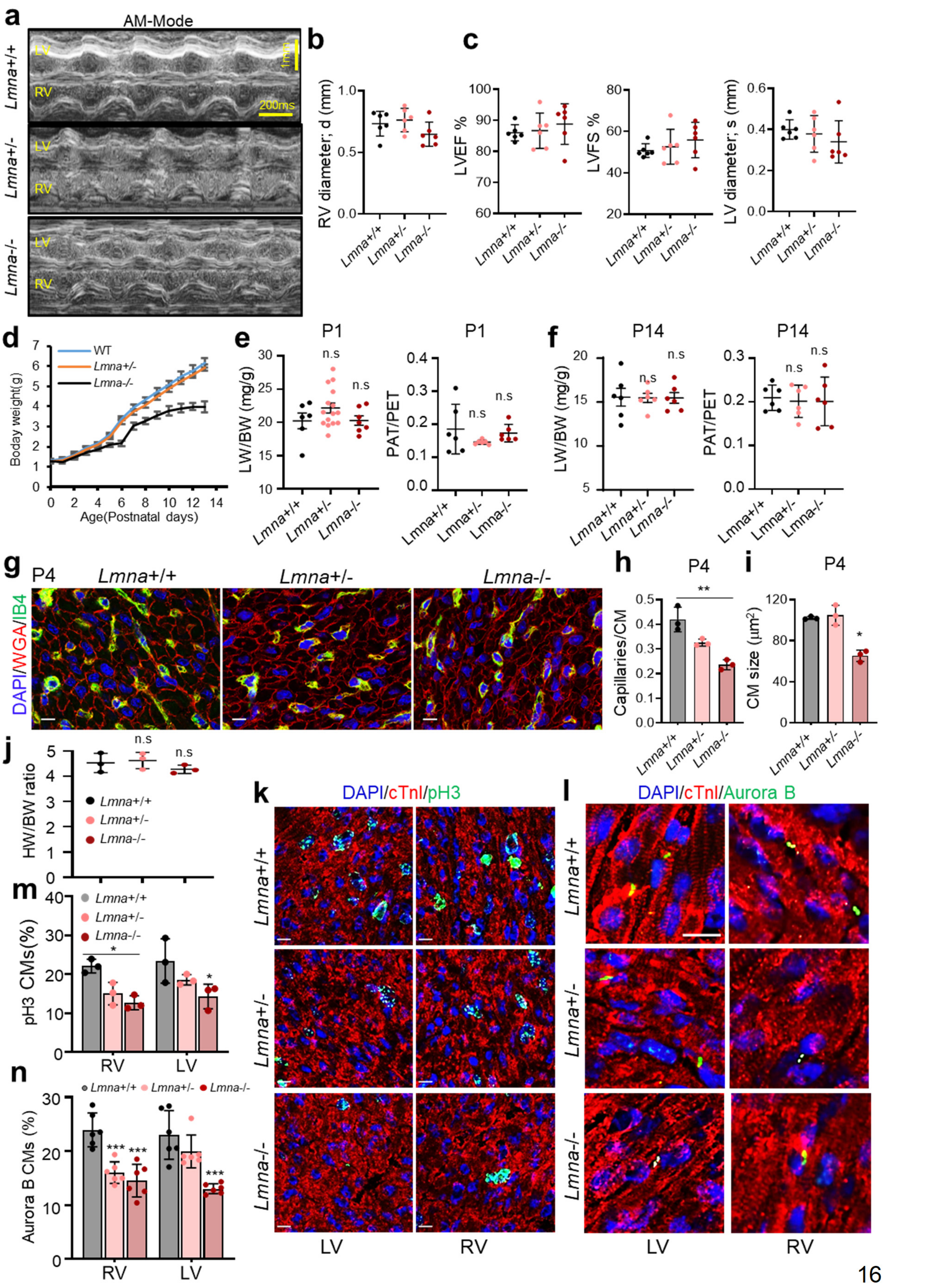
a
AM-Mode
Lmna+/+
Lmna+/-
Lmna-/-
LV
RV
1mm
200ms
b
RV diameter; d (mm)
c
LVEF %
LVFS %
LV diameter; s (mm)
d
WT
Lmna+/-
Lmna-/-
Boday weight(g)
Age(Postnatal days)
e
P1
LW/BW (mg/g)
PAT/PET
n.s
f
P14
g
P4
DAPI/WGA/IB4
h
Capillaries/CM
**
i
CM size (μm2)
*
j
HW/BW ratio
k
DAPI/cTnI/pH3
l
DAPI/cTnI/Aurora B
m
pH3 CMs(%)
RV
LV
n
Aurora B CMs (%)
***

Supplementary Fig. 7: Cardiac phenotype of *Lmna*+/- and *Lmna*-/- Mice.

a Representative examples AM-mode echocardiograms of wild-type, *Lmna*+/- and *Lmna*-/- mice at E16.5. **b** RV diastolic diameter assessed by echocardiography. n=6 mice for each group. **c** Left ventricular ejection fraction (LVEF, left) and left ventricular fractional shortening (LVFS, middle) and LV systolic diameter (right) assessed by echocardiography in wild-type, *Lmna*+/- and *Lmna*-/- embryos at E16.5. n=6 mice for each group. **d** Body weight at the indicated days after birth. n=6 mice for each group. ***e*** Quantification of the lung weight/body weight (LW/BW) ratio of *Lmna*+/+ (n=6), *Lmna*+/- (n=18) and *Lmna*-/- (n=7) mice and pulmonary acceleration time/ pulmonary ejection time (PAT/PET) ratio of *Lmna*+/+ (n=6), *Lmna*+/- (n=6) and *Lmna*-/- (n=6) mice at P1. **f** Quantification of the LW/BW and PAT/PET ratio of *Lmna*+/+, *Lmna*+/- and *Lmna*-/- at P14. n=6 mice for each group. **g** Immunostaining of heart sections with isolectin B4 (IB4, green), wheat germ agglutinin (WGA, red) and DAPI (blue) of *Lmna*+/+, *Lmna*+/- and *Lmna*-/- mice at P4. n=3 mice for each group. Scale bars, 10 µm. **h**, **i** Quantification of the capillaries/CM ratio (**h**) and of the cardiomyocyte cross-sectional area (**I**) of *Lmna*+/+, *Lmna*+/- and *Lmna*-/- mice at P4. n=3 mice for each group. **j** Heart weight/body weight (HW/BW) ratio of *Lmna*+/+, *Lmna*+/- and *Lmna*-/- mice at P4. n=3 mice for each group. **k**, **m** Immunostaining of RV and LV heart sections for phospho-histone H3 (pH3) (green), cardiac troponin I (red) and nucleus (DAPI, blue) at P4 (**k**) and quantification of the percentage of mitotic RV and LV cardiomyocytes at P4 (**m**). n=3 mice for each group. Scale bars, 10 µm. **l**, **n** Immunofluorescent staining of heart sections for aurora B kinase (green), cardiac troponin I (red) and nucleus (DAPI, blue) at P1 (**l**) and quantification of the percentage of CMs in cytokinesis at P1 (**n**). Scale bars, 10 µm. n=6 mice for each group. Data are presented as mean ± SD; One-way ANOVA with Tukey correction (**b-f**, **n**) and Kruskal-Wallis Test with Dunn's correction (**h-j**, **m)** comparisons were used; P-values are as follows: ***p < 0.001, **p < 0.01, *p < 0.05. Source data are provided as a Source Data file.

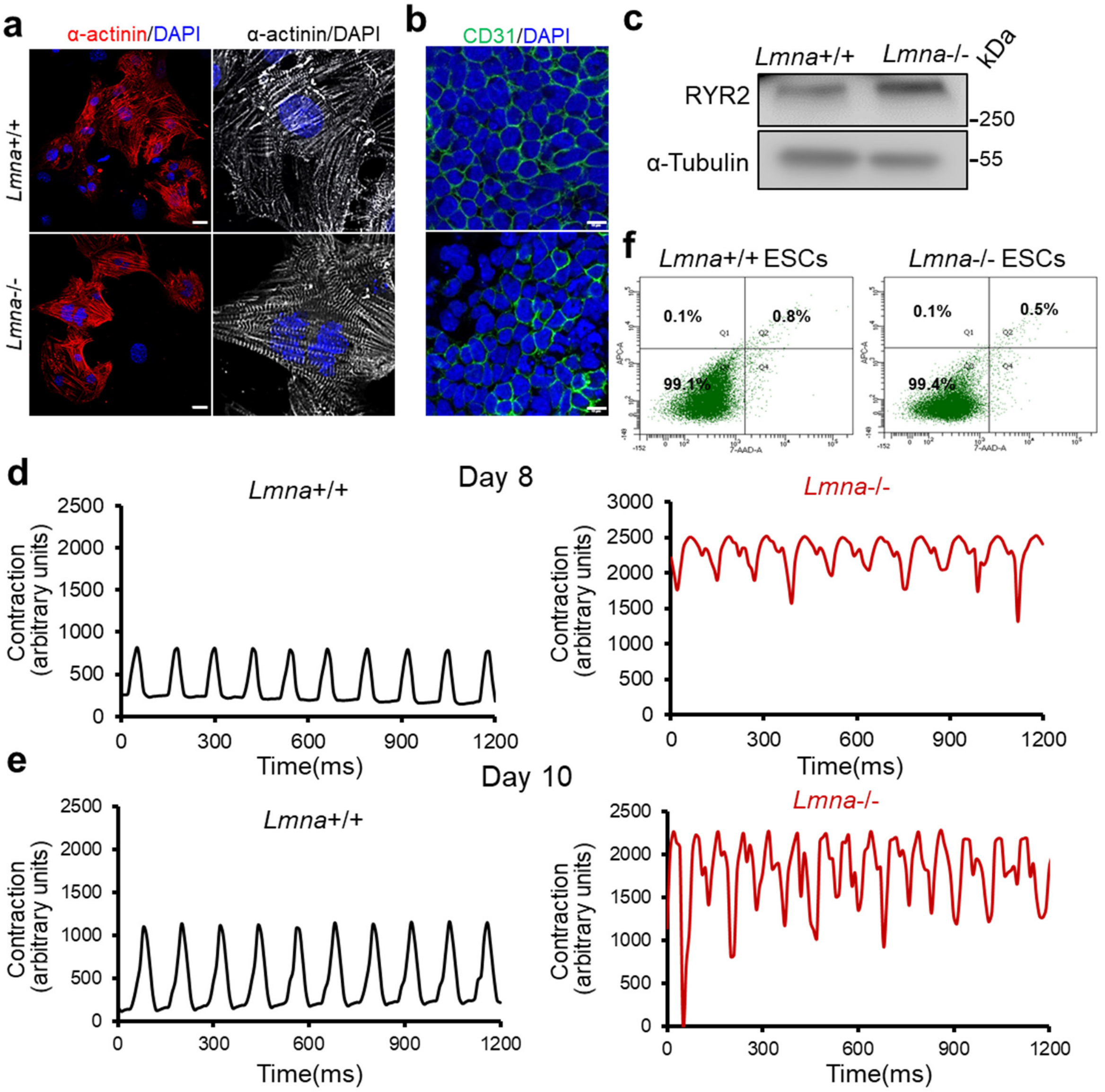

Supplementary Fig. 8: Characterization of control and *Lmna-/-* ESCs and CMs.

a Immunostaining of FACS sorted Nkx2.5+ CMs from day 20 EBs with α-actinin and DAPI. Scale bars, 10 μm. **b** Immunostaining of EC in day 12 EBs usind CD31 antibody. Scale bars, 10 μm. **c** Western blot analysis of Ryr2 in control and *Lmna-/-* CMs. **d**, **e** Representative contraction traces in spontaneously beating CMs at day 8 and day 10 determined by MUSCLEMOTION V1.0. **f** Representative FACS analysis of mESCs stained with Annexin V-APC and 7-AAD.

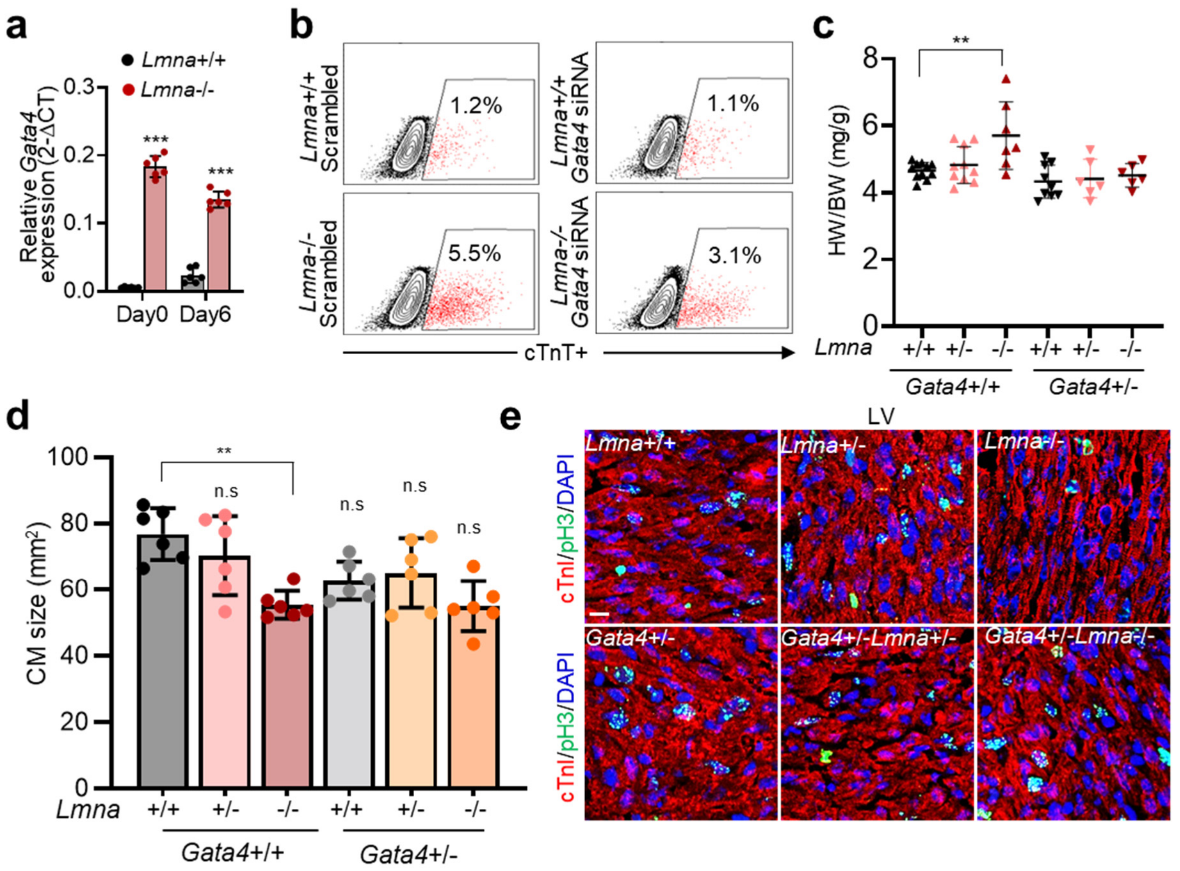

Supplementary Fig. 9: Gata4 downregulation rescues the cardiac phenotype upon lamin A loss.

a Relative *Gata4* mRNA expression in *Lmna*+/+ *and Lmna*-/- ESCs and CPs (n=6). **b** Representative FACS plots of cTnT+ CMs measured in day 10 EBs differentiated after transient knockdown of *Gata4* by siRNA in ESCs*.* **c** Heart weight to body weight ratio (HW/BW) of *Lmna*+/+ (n=11), *Lmna*+/- (n=10), *Lmna*-/- (n=7), *Gata4*+/- (n=9), *Lmna*+/-*Gata4*+/- (n=6) and *Lmna*-/-*Gata4*+/- (n=6) mice at P1. **d** Quantification of CM cross-sectional area in P1 mouse hearts with the indicated genotypes (n=6). **e** Immunostaining of LV heart sections for the mitotic marker phospho-histone H3 (Ser10) (green), cardiac troponin I (red) and nucleus (DAPI, blue) at P1. Scale bars, 10 µm. **a** Data are presented as mean ± SD and p-values were determined by unpaired two-tailed Student's t-test. **c**, **d** Data are presented as mean ± SD and One-way ANOVA with Tukey correction comparisons were used. **a**, **c**, **d** P-values are as follows: ***$p < 0.001$, **$p < 0.01$, *$p < 0.05$. Source data are provided as a Source Data file.

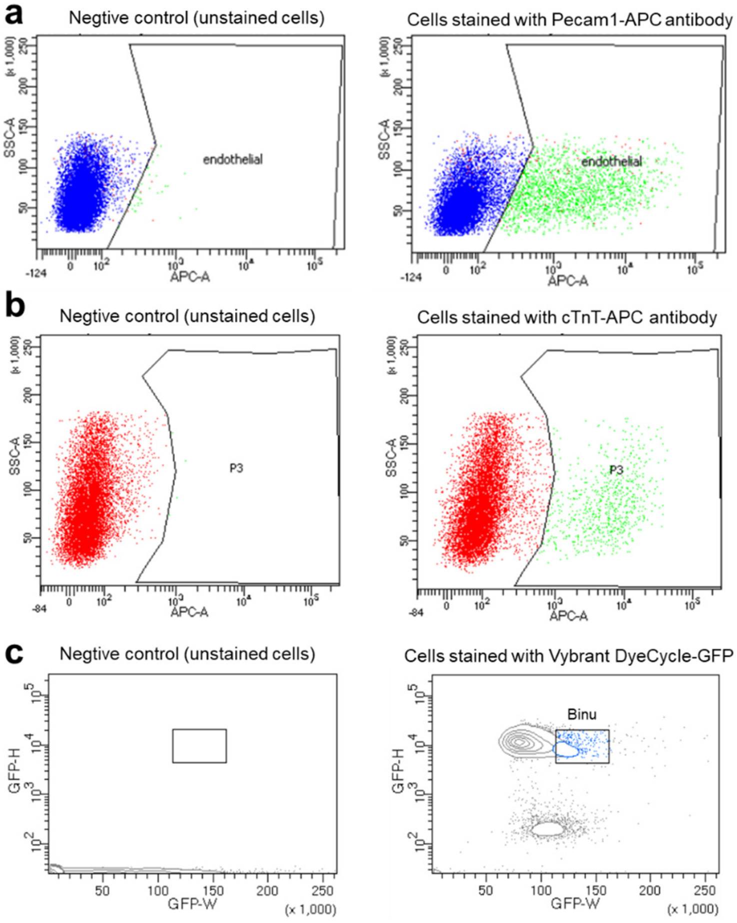

Supplementary Fig. 10: FACS gating strategy. Gates used for unstained cells or cells stained with APC-conjugated anti-Pecam1 (**a**), APC-conjugated anti-troponin T antibodies (**b**) and Vybrant DyeCycle DNA dye (**c**) are presented.

Full unedited gels for Figure S1b

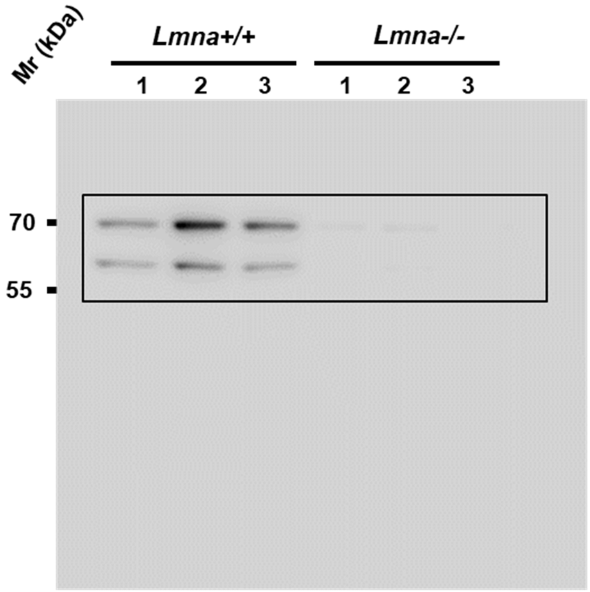

Lamin A/C

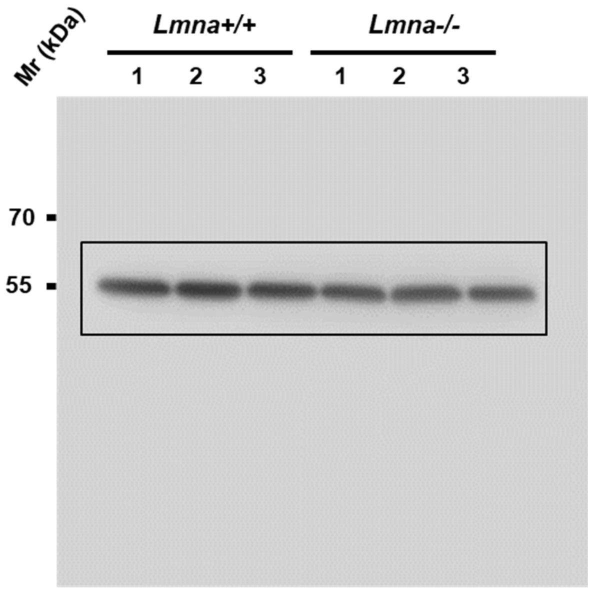

α-Tubulin

Full unedited gels for Figure S2h

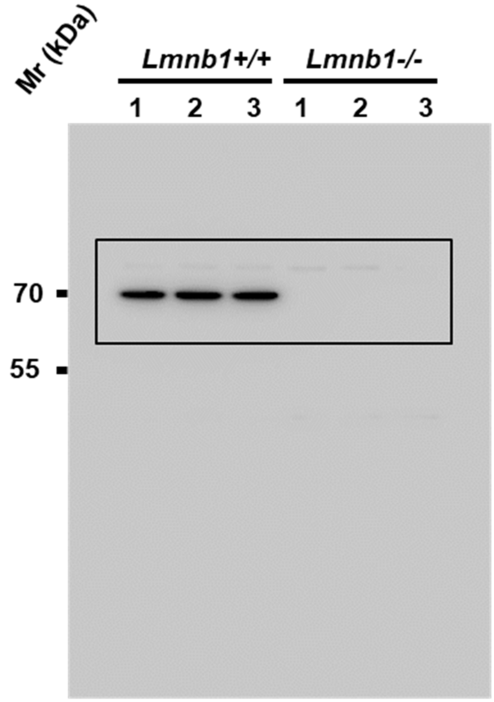

Lamin B1

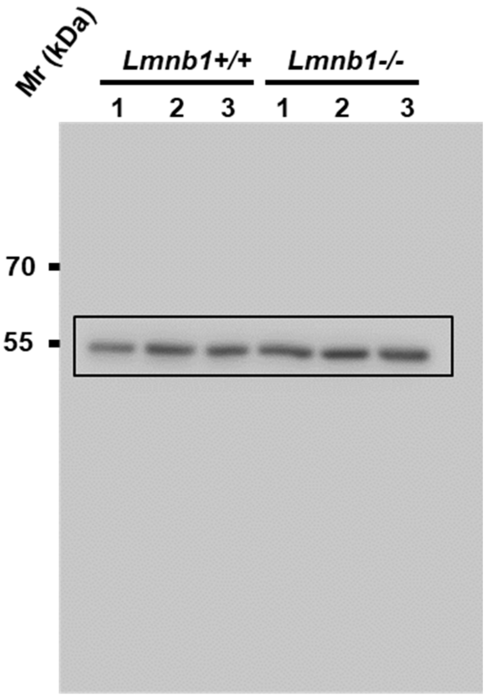

α-Tubulin

Full unedited gels for Figure S4I

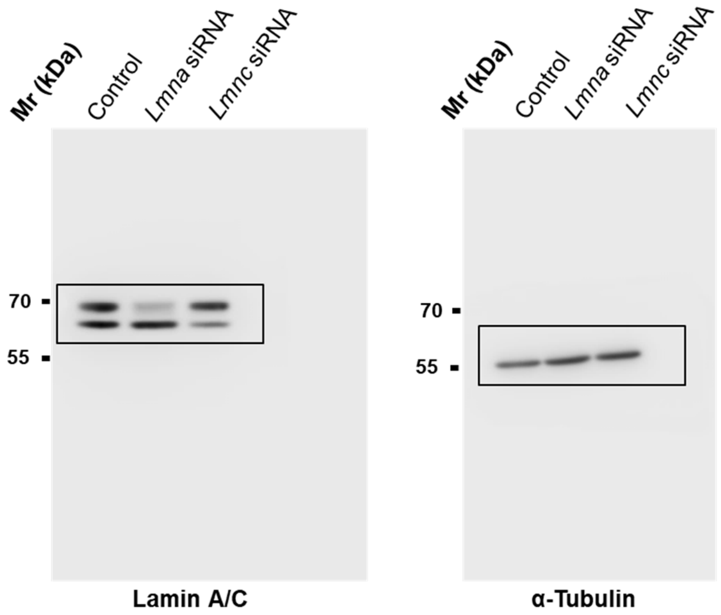

Full unedited gels for Figure S8c

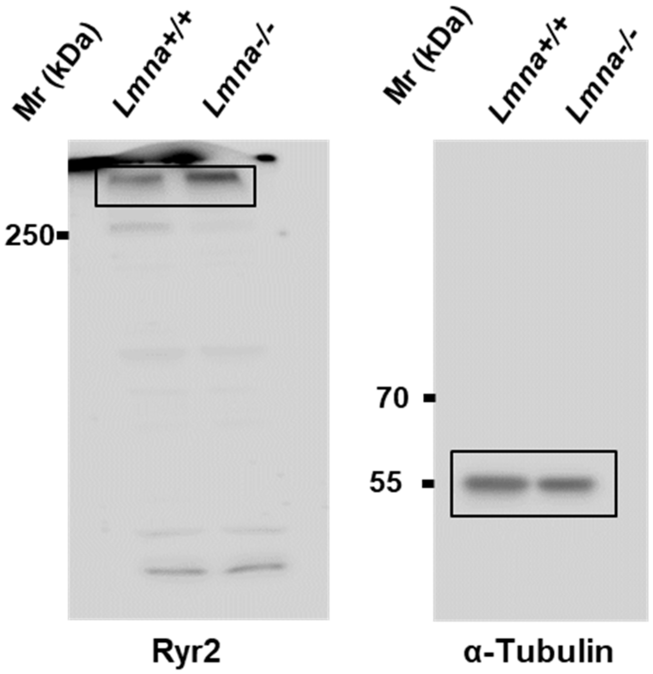

Supplement: Supplementary file 1 — Supplementary Information [file 41467_2022_34366_MOESM1_ESM.pdf]
