# Supplementary material for: Lamin A/C-dependent chromatin architecture safeguards naïve pluripotency to prevent aberrant cardiovascular cell fate and function
Source: Nat Commun. 2022 Nov 4;13:6663. doi: 10.1038/s41467-022-34366-7 (PMC9636150; doi:10.1038/s41467-022-34366-7)
Supplement: Supplementary file 3 — Description of Additional Supplementary Files [file 41467_2022_34366_MOESM3_ESM.pdf]

Description of Additional Supplementary Files

File Name: Supplementary Data 1
Description: RNA-seq Analysis of FACS-sorted CPs, CMs and d10 EBs.

File Name: Supplementary Data 2
Description: Hi-C Analysis of A/B Compartment Transitions in ESCs

File Name: Supplementary Data 3
Description: RNA-seq Analysis of mESCs.

File Name: Supplementary Data 4
Description: ATAC-Seq Peaks in mESCs.

File Name: Supplementary Data 5
Description: ATAC-Seq Peaks in FACS-sorted CMs from Day 10 EBs.

File Name: Supplementary Data 6
Description: Antibodies.

File Name: Supplementary Data 7
Description: Programs and algorithms.

File Name: Supplementary Movie 1
Description: Beating control CMs on day 8 of directed cardiomyocyte differentiation.

File Name: Supplementary Movie 2
Description: Beating Lmna−/−CMs on day 8 of directed cardiomyocyte differentiation.

File Name: Supplementary Movie 3
Description: Beating control CMs on day 10 of directed cardiomyocyte differentiation.

File Name: Supplementary Movie 4
Description: Beating Lmna−/−CMs on day 10 of directed cardiomyocyte differentiation.
