# Supplementary material for: Lamin A/C-dependent chromatin architecture safeguards naïve pluripotency to prevent aberrant cardiovascular cell fate and function
Source: Nat Commun. 2022 Nov 4;13:6663. doi: 10.1038/s41467-022-34366-7 (PMC9636150; doi:10.1038/s41467-022-34366-7)
Supplement: Supplementary file 9 — Supplementary Data 6 [file 41467_2022_34366_MOESM9_ESM.pdf]

Supplementary Table 6. Antibodies.

Antibody	Source	Catalogue number
Mouse monoclonal anti-LaminA/C (E-1)	Santa Cruz	Cat# sc-376248, RRID:AB_10991536
Mouse monoclonal anti-LaminA/C (131C3)	Abcam	Cat# ab8984, RRID:AB_306913
Rabbit polyclonal anti-LaminB1	Abcam	Cat# ab16048, RRID:AB_10107828
Rabbit polyclonal anti-LaminB1	Sigma	Cat# HPA050524, RRID:AB_2681156
Mouse monoclonal anti-LaminB1 (B10)	Santa Cruz	Cat# sc-374015
Goat polyclonal anti-Gata4(C-20)	Santa Cruz	Cat# sc-1237, RRID:AB_2108747
Mouse monoclonal anti-OCT3/4 (C-10)	Santa Cruz	Cat# sc-5279, RRID:AB_628051
Rabbit polyclonal anti-MYL4	Sigma	Cat# HPA051884, RRID:AB_2681651
Goat polyclonal anti-Cardiac Troponin I	Abcam	Cat# ab56357, RRID:AB_880622
Mouse monoclonal Anti-phospho-Histone H2A.X (Ser139)	Millipore	Cat# 05-636-I, RRID:AB_2755003
CD31 (PECAM-1) Monoclonal Antibody (390), APC, eBioscience™	Thermo Fisher Scientific	Cat# 17-0311-82, RRID:AB_657735
Alexa Fluor® 647 Mouse Anti-Cardiac Troponin T Clone 13-11	BD Biosciences	Cat# 565744, RRID:AB_2739341
Wheat germ agglutinin, Alexa Fluor®488 conjugate	Thermo Fisher Scientific	Cat# W11261
Isolectin GS-IB4 from Griffonia simplicifolia, Alexa Fluor® 568 conjugate	Thermo Fisher Scientific	Cat# 121412
CD309 (FLK1) Monoclonal Antibody (Avas12a1), APC, eBioscience™	Thermo Fisher Scientific	Cat# 17-5821-81, RRID:AB_657866
CD140a (PDGFRA) Monoclonal Antibody (APA5), PE, eBioscience™	Thermo Fisher Scientific	Cat# 12-1401-81, RRID:AB_657615
Rabbit polyclonal anti-phospho-Histone H3 (Ser10)	Millipore	Cat# 06-570, RRID:AB_310177
Rabbit polyclonal anti-Ryr2	Sigma	Cat# HPA020028, RRID:AB_1856528
Mouse monoclonal anti-alpha-Tubulin clone 2-28-33	Sigma	Cat# T5168, RRID:AB_477579
Sheep polyclonal anti-Digoxigenin-Rhodamine, Fab fragments	Roche	Cat# 11207750910, RRID:AB_514501
Rabbit polyclonal anti-Aurora B	Abcam	Cat# ab2254, RRID:AB_302923
Donkey anti-Mouse IgG (H+L) ReadyProbes™ Secondary Antibody, Alexa Fluor 488	Thermo Fisher Scientific	Cat# R37114, RRID:AB_2556542
Donkey anti-Mouse IgG (H+L) Highly Cross-Adsorbed Secondary Antibody, Alexa Fluor 555	Thermo Fisher Scientific	Cat# A-31570, RRID:AB_2536180
Donkey anti-Goat IgG (H+L) Cross-Adsorbed Secondary Antibody, Alexa Fluor 555	Thermo Fisher Scientific	Cat# A-21432, RRID:AB_2535853
Donkey anti-Rabbit IgG (H+L) Highly Cross-Adsorbed Secondary Antibody, Alexa Fluor 488	Thermo Fisher Scientific	Cat# A-21206, RRID:AB_2535792
Donkey anti-Goat IgG (H+L) Cross-Adsorbed Secondary Antibody, Alexa Fluor 488	Thermo Fisher Scientific	Cat# A-11055, RRID:AB_2534102
