# Supplementary material for: Lamin A/C-dependent chromatin architecture safeguards naïve pluripotency to prevent aberrant cardiovascular cell fate and function
Source: Nat Commun. 2022 Nov 4;13:6663. doi: 10.1038/s41467-022-34366-7 (PMC9636150; doi:10.1038/s41467-022-34366-7)
Supplement: Supplementary file 10 — Supplementary Data 7 [file 41467_2022_34366_MOESM10_ESM.pdf]

Supplementary Table 7. Programs and algorithms.

Programs and algorithms	Source	Website
IGV2.8.13	Integrative Genomics Viewer	https://software.broadinstitute.org/software/igv/download
Image J 1.47v	Image J	https://imagej.nih.gov/ij/download.html
Vevo LAB Software Package V3.2.6	FUJIFILM VisualSonics	https://www.visualsonics.com/resource/vevo-lab-software
IonWizard 7.4	IonOptix	https://www.ionoptix.com/
MUSCLEMOTION V1.0	[1]	https://github.com/l-sala/MUSCLEMOTION
BD FACSDiva Software (version8.0.1, firmware version 1.49 BD FACSCanto II).	BD Biosciences	https://www.bdbiosciences.com/en-eu/products/software/instrument-software/bd-facsdiva-software
Zen 2.3	ZEISS	https://www.zeiss.de/mikroskopie/produkte/mikroskopsoftware/zen-lite/zen-lite-download.html
DAVID 6.8	[2]	https://david.ncifcrf.gov/summary.jsp
Heatmapper	[3]	http://www.heatmapper.ca/expression/
GraphPad Prism 8.0.2	GraphPad	https://www.graphpad.com/
WashU Epigenome Browser	[4]	http://epigenomegateway.wustl.edu/
Calculate and draw custom Venn diagrams	Bioinformatics & Evolutionary Genomics	http://bioinformatics.psb.ugent.be/webtools/Venn/
STAR version 2.7.3a	[5]	https://github.com/alexdobin/STAR/blob/master/doc/STARmanual.pdf
Trimmomatic version 0.39	[6]	http://www.usadellab.org/cms/?page=trimmomatic
BamTools version 2.5.1	[7]	https://github.com/pezmaster31/bamtools
MultiQC version 1.6	[8]	https://multiqc.info/
DESeq2 version 1.28.0	[9]	http://bioconductor.org/packages/release/bioc/vignettes/DESeq2/inst/doc/DESeq2.html

Ngsplot version 2.41.4	10	https://github.com/shenlab-sinai/ngsplot
Homer version 4.11	11	http://homer.ucsd.edu/homer/motif/
Bowtie2 version 2.3.4.1	12	https://github.com/BenLangmead/bowtie2
SAMtools version 1.7	13	http://www.htslib.org/
Picard-tools version 1.119		https://broadinstitute.github.io/picard/
deepTools version 3.3.0	14	https://deeptools.readthedocs.io/en/develop/
MACS2 version 2.1.1.20160309	Gaspar, 2018	https://pypi.org/project/MACS2/
Bedtools version 2.28.0	15	https://bedtools.readthedocs.io/en/latest/
R package DiffBind version 2.16.0	16	http://bioconductor.org/packages/release/bioc/vignettes/DiffBind/inst/doc/DiffBind.pdf
R package ChIPseeker version 1.24.0	17	https://guangchuangyu.github.io/software/ChIPseeker/
R package rtracklayer version 1.48.0	18	https://bioconductor.org/packages/release/bioc/html/rtracklayer.html
R package EnhancedVolcano version 1.6.0	Blighe K et al, 2020	https://github.com/kevinblighe/EnhancedVolcano
HiC-Pro2.1.1.4	Github, Servant N et al.,2015	https://github.com/nservant/HiC-Pro
FitHiChIP 8.1	19	https://ay-lab.github.io/FitHiChIP/
HiCExplorer 3.6	20	https://hicexplorer.readthedocs.io/en/latest/

References:

1 Sala, L. *et al.* MUSCLEMOTION: A Versatile Open Software Tool to Quantify Cardiomyocyte and Cardiac Muscle Contraction In Vitro and In Vivo. *Circ Res* **122**, e5-e16, doi:10.1161/CIRCRESAHA.117.312067 (2018).

2 Huang da, W., Sherman, B. T. & Lempicki, R. A. Systematic and integrative analysis of large gene lists using DAVID bioinformatics resources. *Nat Protoc* **4**, 44-57, doi:10.1038/nprot.2008.211 (2009).

3 Babicki, S. *et al.* Heatmapper: web-enabled heat mapping for all. *Nucleic Acids Res* **44**, W147-153, doi:10.1093/nar/gkw419 (2016).

4 Zhou, X. *et al.* The Human Epigenome Browser at Washington University. *Nat Methods* **8**, 989-990, doi:10.1038/nmeth.1772 (2011).

5 Dobin, A. *et al.* STAR: ultrafast universal RNA-seq aligner. *Bioinformatics* **29**, 15-21, doi:10.1093/bioinformatics/bts635 (2013).

6 Bolger, A. M., Lohse, M. & Usadel, B. Trimmomatic: a flexible trimmer for Illumina sequence data. *Bioinformatics* **30**, 2114-2120, doi:10.1093/bioinformatics/btu170 (2014).

7 Barnett, D. W., Garrison, E. K., Quinlan, A. R., Stromberg, M. P. & Marth, G. T. BamTools: a C++ API and toolkit for analyzing and managing BAM files. *Bioinformatics* **27**, 1691-1692, doi:10.1093/bioinformatics/btr174 (2011).

8 Ewels, P., Magnusson, M., Lundin, S. & Kaller, M. MultiQC: summarize analysis results for multiple tools and samples in a single report. *Bioinformatics* **32**, 3047-3048, doi:10.1093/bioinformatics/btw354 (2016).

9 Love, M. I., Huber, W. & Anders, S. Moderated estimation of fold change and dispersion for RNA-seq data with DESeq2. *Genome Biol* **15**, 550, doi:10.1186/s13059-014-0550-8 (2014).

10 Shen, L., Shao, N., Liu, X. & Nestler, E. ngs.plot: Quick mining and visualization of next-generation sequencing data by integrating genomic databases. *BMC Genomics* **15**, 284, doi:10.1186/1471-2164-15-284 (2014).

11 Heinz, S. *et al.* Simple combinations of lineage-determining transcription factors prime cis-regulatory elements required for macrophage and B cell identities. *Mol Cell* **38**, 576-589, doi:10.1016/j.molcel.2010.05.004 (2010).

12 Langmead, B. & Salzberg, S. L. Fast gapped-read alignment with Bowtie 2. *Nat Methods* **9**, 357-359, doi:10.1038/nmeth.1923 (2012).

13 Li, H. *et al.* The Sequence Alignment/Map format and SAMtools. *Bioinformatics* **25**, 2078-2079, doi:10.1093/bioinformatics/btp352 (2009).

14 Ramirez, F. *et al.* deepTools2: a next generation web server for deep-sequencing data analysis. *Nucleic Acids Res* **44**, W160-165, doi:10.1093/nar/gkw257 (2016).

15 Quinlan, A. R. & Hall, I. M. BEDTools: a flexible suite of utilities for comparing genomic features. *Bioinformatics* **26**, 841-842, doi:10.1093/bioinformatics/btq033 (2010).

16 Ross-Innes, C. S. *et al.* Differential oestrogen receptor binding is associated with clinical outcome in breast cancer. *Nature* **481**, 389-U177, doi:10.1038/nature10730 (2012).

17 Yu, G., Wang, L. G. & He, Q. Y. ChIPseeker: an R/Bioconductor package for ChIP peak annotation, comparison and visualization. *Bioinformatics* **31**, 2382-2383, doi:10.1093/bioinformatics/btv145 (2015).

18 Lawrence, M., Gentleman, R. & Carey, V. rtracklayer: an R package for interfacing with genome browsers. *Bioinformatics* **25**, 1841-1842, doi:10.1093/bioinformatics/btp328 (2009).

19 Bhattacharyya, S., Chandra, V., Vijayanand, P. & Ay, F. Identification of significant chromatin contacts from HiChIP data by FitHiChIP. *Nat Commun* **10**, 4221, doi:10.1038/s41467-019-11950-y (2019).
20 Ramirez, F. *et al.* High-resolution TADs reveal DNA sequences underlying genome organization in flies. *Nat Commun* **9**, 189, doi:10.1038/s41467-017-02525-w (2018).
