# Supplementary material for: Lamin A/C-dependent chromatin architecture safeguards naïve pluripotency to prevent aberrant cardiovascular cell fate and function
Source: Nat Commun. 2022 Nov 4;13:6663. doi: 10.1038/s41467-022-34366-7 (PMC9636150; doi:10.1038/s41467-022-34366-7)
Supplement: Supplementary file 15 — Reporting Summary [file 41467_2022_34366_MOESM15_ESM.pdf]

Corresponding author(s): Gergana Dobreva

Last updated by author(s): Oct 3, 2022

Reporting Summary

Nature Research wishes to improve the reproducibility of the work that we publish. This form provides structure for consistency and transparency in reporting. For further information on Nature Research policies, see our Editorial Policies and the Editorial Policy Checklist.

Statistics

For all statistical analyses, confirm that the following items are present in the figure legend, table legend, main text, or Methods section.

n/a	Confirmed	
☐	☒	The exact sample size (*n*) for each experimental group/condition, given as a discrete number and unit of measurement
☐	☒	A statement on whether measurements were taken from distinct samples or whether the same sample was measured repeatedly
☐	☒	The statistical test(s) used AND whether they are one- or two-sided<br>*Only common tests should be described solely by name; describe more complex techniques in the Methods section.*
☒	☐	A description of all covariates tested
☐	☒	A description of any assumptions or corrections, such as tests of normality and adjustment for multiple comparisons
☐	☒	A full description of the statistical parameters including central tendency (e.g. means) or other basic estimates (e.g. regression coefficient) AND variation (e.g. standard deviation) or associated estimates of uncertainty (e.g. confidence intervals)
☐	☒	For null hypothesis testing, the test statistic (e.g. *F*, *t*, *r*) with confidence intervals, effect sizes, degrees of freedom and *P* value noted<br>*Give P values as exact values whenever suitable.*
☒	☐	For Bayesian analysis, information on the choice of priors and Markov chain Monte Carlo settings
☒	☐	For hierarchical and complex designs, identification of the appropriate level for tests and full reporting of outcomes
☒	☐	Estimates of effect sizes (e.g. Cohen's *d*, Pearson's *r*), indicating how they were calculated

Our web collection on statistics for biologists contains articles on many of the points above.

Software and code

Policy information about availability of computer code

Data collection	The following standard software provided by instrument suppliers was used for data collection:<br>Calcium transients: MyoPacer (IonOptix)<br>Histology: Zeiss Axio Scan (Ziess)<br>Flow cytometry: BD FACSCanto II<br>WB: Amersham Imager 600(GE Healthcare Life Sciences)<br>Echocardiography: Vevo 3100 high-resolution system (Visualsonics, Toronto, ON)<br>Immunofluorescence : Leica DMi8 microscope (Leica); Zeiss LSM 710 confocal microscope(Zeiss)
Data analysis	IGV2.8.13, https://software.broadinstitute.org/software/igv/download<br>Image J 1.47v, https://imagej.nih.gov/ij/download.html<br>Vevo LAB Software Package V3.2.6, https://www.visualsonics.com/resource/vevo-lab-software<br>IonWizard 7.4, https://www.ionoptix.com/<br>MUSCLEMOTION V1.0, https://github.com/l-sala/MUSCLEMOTION<br>BD FACSDiva Software (version8.0.1, firmware version 1.49 BD FACSCanto II), https://www.bdbiosciences.com/en-eu/products/software/instrument-software/bd-facsdiva-software<br>Zen 2.3, https://www.zeiss.de/mikroskopie/produkte/mikroskopsoftware/zen-lite/zen-lite-download.html<br>DAVID 6.8, https://david.ncifcrf.gov/summary.jsp<br>Heatmapper, http://www.heatmapper.ca/expression/<br>GraphPad Prism 8.0.2, https://www.graphpad.com/<br>WashU Epigenome Browser, http://epigenomegateway.wustl.edu/<br>Calculate and draw custom Venn diagrams, http://bioinformatics.psb.ugent.be/webtools/Venn/<br>STAR version 2.7.3a, https://github.com/alexdobin/STAR/blob/master/doc/STARmanual.pdf<br>Trimmomatic version 0.39, http://www.usadellab.org/cms/?page=trimmomatic<br>BamTools version 2.5.1, https://github.com/pezmaster31/bamtools

MultiQC version 1.6, https://multiqc.info/
DESeq2 version 1.28.0, http://bioconductor.org/packages/release/bioc/vignettes/DESeq2/inst/doc/DESeq2.html
Ngsplot version 2.41.4, https://github.com/shenlab-sinai/ngsplot
Homer version 4.11, http://homer.ucsd.edu/homer/motif/
Bowtie2 version 2.3.4.1 , https://github.com/BenLangmead/bowtie2
SAMtools version 1.7, http://www.htslib.org/
Picard-tools version 1.119, https://broadinstitute.github.io/picard/
deepTools version 3.3.0, https://deeptools.readthedocs.io/en/develop/
MACS2 version 2.1.1.20160309, https://pypi.org/project/MACS2/
Bedtools version 2.28.0 , https://bedtools.readthedocs.io/en/latest/
R package DiffBind version 2.16.0, http://bioconductor.org/packages/release/bioc/vignettes/DiffBind/inst/doc/DiffBind.pdf
R package ChIPseeker version 1.24.0, https://guangchuangyu.github.io/software/ChIPseeker/
R package rtracklayer version 1.48.0, https://bioconductor.org/packages/release/bioc/html/rtracklayer.html
R package EnhancedVolcano version 1.6.0, https://github.com/kevinblighe/EnhancedVolcano
HiC-Pro2.1.1.4, https://github.com/nservant/HiC-Pro
FitHiChIP 8.1, https://ay-lab.github.io/FitHiChIP/
HiCExplorer 3.6, https://hicexplorer.readthedocs.io/en/latest/

For manuscripts utilizing custom algorithms or software that are central to the research but not yet described in published literature, software must be made available to editors and reviewers. We strongly encourage code deposition in a community repository (e.g. GitHub). See the Nature Research guidelines for submitting code & software for further information.

Data

Policy information about availability of data

All manuscripts must include a data availability statement. This statement should provide the following information, where applicable:

- Accession codes, unique identifiers, or web links for publicly available datasets
- A list of figures that have associated raw data
- A description of any restrictions on data availability

Raw RNA-seq, ATAC-seq and Hi-C data generated in this study have been deposited in GEO database under accession code GSE164069. Processed RNA-seq, ATAC-seq and Hi-C data are provided in the Supplementary Information. Lamin A DamID (GSE62685) and Lamin B1 DamID data (GSE17051), expression data during early mouse (GSE57249) and human embryonic development (GSE36552 and GSE101571), mESC, mESC-CMs (GSE47948), hiPSC and hiPSC-CM (GSE107654) as well as patients with LMNA-associated DCM mutation (GSE120836) were retrieved from previously published studies. Source data are provided with this paper.

Field-specific reporting

Please select the one below that is the best fit for your research. If you are not sure, read the appropriate sections before making your selection.

☒ Life sciences ☐ Behavioural & social sciences ☐ Ecological, evolutionary & environmental sciences

For a reference copy of the document with all sections, see nature.com/documents/nr-reporting-summary-flat.pdf

Life sciences study design

All studies must disclose on these points even when the disclosure is negative.

Sample size	No statistical measures were used to determine sample size. Sample size is based on our experience and on publications by other groups. (e.g.Nature communications, 2018, 9(1): 1-16;Cell research, 2019, 29(6): 486-501)
Data exclusions	None.
Replication	All experiments were performed at least three independent times. All attempts at replication were successful.
Randomization	Mice were randomly allocated into experimental groups.
Blinding	The investigators were not blinded in regard to allocation of samples during experiments and outcome assessment.However, the outcomes were quantitative and not subjective.

Reporting for specific materials, systems and methods

We require information from authors about some types of materials, experimental systems and methods used in many studies. Here, indicate whether each material, system or method listed is relevant to your study. If you are not sure if a list item applies to your research, read the appropriate section before selecting a response.

Materials & experimental systems

n/a	Involved in the study
☐	☒ Antibodies
☐	☒ Eukaryotic cell lines
☒	☐ Palaeontology and archaeology
☐	☒ Animals and other organisms
☒	☐ Human research participants
☒	☐ Clinical data
☒	☐ Dual use research of concern

Methods

n/a	Involved in the study
☐	☒ ChIP-seq
☐	☒ Flow cytometry
☒	☐ MRI-based neuroimaging

Antibodies

Antibodies used

Mouse monoclonal anti-LaminA/C (E-1), Santa Cruz,Cat# sc-376248, RRID:AB_10991536, 1:100 for IF and 1:1000 for WB
Mouse monoclonal anti-LaminA/C (131C3), Abcam, Cat# ab8984, RRID:AB_306913, 1:100 for IF and 1:1000 for WB
Rabbit polyclonal anti-LaminB1, Abcam, Cat# ab16048, RRID:AB_10107828, 1:100 for IF and 1:1000 for WB
Rabbit polyclonal anti-LaminB1, Sigma, Cat# HPA050524, RRID:AB_2681156, 1:100 for IF and 1:1000 for WB
Mouse monoclonal anti-LaminB1 (B10) , Santa Cruz, Cat# sc-374015, 1:100 for IF and 1:1000 for WB
Goat polyclonal anti-Gata4(C-20), Santa Cruz, Cat# sc-1237, RRID:AB_2108747, 1:100
Mouse monoclonal anti-OCT3/4 (C-10) , Santa Cruz, Cat# sc-5279, RRID:AB_628051, 1:100
Rabbit polyclonal anti-MYL4, Sigma, Cat# HPA051884, RRID:AB_2681651, 1:100
Goat polyclonal anti-Cardiac Troponin I, Abcam, Cat# ab56357, RRID:AB_880622, 1:100
Mouse monoclonal Anti-phospho-Histone H2A.X (Ser139), Millipore, Cat# 05-636-I, RRID:AB_2755003, 1:100 for IF and 1:1000 for WB
CD31 (PECAM-1) Monoclonal Antibody (390), APC, eBioscience™, Thermo Fisher Scientific, Cat# 17-0311-82, RRID:AB_657735, 1:40
Alexa Fluor® 647 Mouse Anti-Cardiac Troponin T Clone 13-11, BD Biosciences, Cat# 565744, RRID:AB_2739341, 1:40
Wheat germ agglutinin, Alexa Fluor®488 conjugate, Thermo Fisher Scientific, Cat# W11261, 1:100
Isolectin GS-IB4 from Griffonia simplicifolia, Alexa Fluor® 568 conjugate , Thermo Fisher Scientific, Cat# 121412, 1:100
CD309 (FLK1) Monoclonal Antibody (Avas12a1), APC, eBioscience™, Thermo Fisher Scientific, Cat# 17-5821-81, RRID:AB_657866, 1:40
CD140a (PDGFRA) Monoclonal Antibody (APA5), PE, eBioscience™, Thermo Fisher Scientific, Cat# 12-1401-81, RRID:AB_657615, 1:40
Rabbit polyclonal anti-phospho-Histone H3 (Ser10), Millipore, Cat# 06-570, RRID:AB_310177, 1:40
Rabbit polyclonal anti-Ryr2, Sigma, Cat# HPA020028, RRID:AB_1856528, 1:1000
Mouse monoclonal anti-alpha-Tubulin clone 2-28-33, Sigma, Cat# T5168, RRID:AB_477579, 1:1000
Sheep polyclonal anti-Digoxigenin-Rhodamine, Fab fragments, Roche, Cat# 11207750910, RRID:AB_514501, 1:250
Rabbit polyclonal anti-Aurora B , Abcam, Cat# ab2254, RRID:AB_302923, 1:100
Donkey anti-Mouse IgG (H+L) ReadyProbes™ Secondary Antibody, Alexa Fluor 488, Thermo Fisher Scientific, Cat# R37114, RRID:AB_2556542, 1:500
Donkey anti-Mouse IgG (H+L) Highly Cross-Adsorbed Secondary Antibody, Alexa Fluor 555, Thermo Fisher Scientific , Cat# A-31570, RRID:AB_2536180, 1:500
Donkey anti-Goat IgG (H+L) Cross-Adsorbed Secondary Antibody, Alexa Fluor 555, Thermo Fisher Scientific, Cat# A-21432, RRID:AB_2535853, 1:500
Donkey anti-Rabbit IgG (H+L) Highly Cross-Adsorbed Secondary Antibody, Alexa Fluor 488, Thermo Fisher Scientific , Cat# A-21206, RRID:AB_2535792, 1:500
Donkey anti-Goat IgG (H+L) Cross-Adsorbed Secondary Antibody, Alexa Fluor 488, Thermo Fisher Scientific, Cat# A-11055, RRID:AB_2534102, 1:500

Validation

All antibodies used were purchased from commercial vendors and were selected because they have been validated by the manufacturer and in different publications. Validation details and relevant publications are detailed on their respective websites.
Anti-Lamin A/C Antibody (E-1): https://www.scbt.com/p/lamin-a-c-antibody-e-1?productCanUrl=lamin-a-c-antibody-e-1&_requestid=793642
Mouse monoclonal anti-LaminA/C (131C3): https://www.abcam.com/lamin-a--lamin-c-antibody-131c3-nuclear-envelope-marker-ab8984.html
Rabbit polyclonal anti-LaminB1: https://www.abcam.com/lamin-b1-antibody-nuclear-envelope-marker-ab16048.html
Rabbit polyclonal anti-LaminB1: https://www.sigmaaldrich.com/DE/en/product/sigma/hpa050524
Mouse monoclonal anti-LaminB1 (B10): https://www.scbt.com/p/lamin-b1-antibody-b-10
Goat polyclonal anti-Gata4(C-20): https://www.scbt.com/p/gata-4-antibody-c-20
Mouse monoclonal anti-OCT3/4 (C-10): https://www.scbt.com/p/oct-3-4-antibody-c-10?productCanUrl=oct-3-4-antibody-c-10&_requestid=798972
Rabbit polyclonal anti-MYL4: https://www.proteinatlas.org/ENSG00000198336-MYL4/antibody
Goat polyclonal anti-Cardiac Troponin I: https://www.abcam.com/cardiac-troponin-i-antibody-ab56357.html
Mouse monoclonal Anti-phospho-Histone H2A.X (Ser139):
https://www.merckmillipore.com/DE/de/product/Anti-phospho-Histone-H2A.X-Ser139-Antibody-clone-JBW301,MM_NF-05-636?ReferrerURL=https%3A%2F%2Fwww.google.com%2F
CD31 (PECAM-1) Monoclonal Antibody (390), APC, eBioscience™: https://www.thermofisher.com/antibody/product/CD31-PECAM-1-Antibody-clone-390-Monoclonal/17-0311-82
Alexa Fluor® 647 Mouse Anti-Cardiac Troponin T Clone 13-11: https://www.bdbiosciences.com/en-nz/products/reagents/flow-cytometry-reagents/research-reagents/single-color-antibodies-ruo/alexa-fluor-647-mouse-anti-cardiac-troponin-t.565744
Wheat germ agglutinin, Alexa Fluor®488 conjugate: https://www.thermofisher.com/order/catalog/product/W11261
Isolectin GS-IB4 from Griffonia simplicifolia, Alexa Fluor® 568 conjugate: https://www.thermofisher.com/order/catalog/product/

I21412
CD309 (FLK1) Monoclonal Antibody (Avas12a1), APC, eBioscience™: https://www.thermofisher.com/antibody/product/CD309-FLK1-Antibody-clone-Avas12a1-Monoclonal/17-5821-81
CD140a (PDGFRA) Monoclonal Antibody (APA5), PE, eBioscience™: https://www.thermofisher.com/antibody/product/CD140a-PDGFRA-Antibody-clone-APA5-Monoclonal/12-1401-81
Rabbit polyclonal anti-phospho-Histone H3 (Ser10): https://www.merckmillipore.com/DE/de/product/Anti-phospho-Histone-H3-Ser10-Antibody-Mitosis-Marker,MM_NF-06-570?ReferrerURL=https%3A%2F%2Fwww.google.com%2F

Rabbit polyclonal anti-Ryr2: https://www.sigmaaldrich.com/DE/en/product/sigma/hpa020028
Mouse monoclonal anti-alpha-Tubulin(B-5-1-2): https://www.sigmaaldrich.com/DE/en/product/sigma/t5168
Sheep polyclonal anti-Digoxigenin-Rhodamine, Fab fragments: https://www.sigmaaldrich.com/DE/en/product/roche/11207750910
Rabbit polyclonal anti-Aurora B: https://www.abcam.com/Aurora-B-antibody-ab2254.html?gclsrc=aw.ds|aw.ds&gclid=EAIaIQobChMIl4at2dDE-gIVEwOLCh3RnwvlEAAYASAAEgKJ0fD_BwE
Donkey anti-Mouse IgG (H+L) ReadyProbes™ Secondary Antibody, Alexa Fluor 488: https://www.thermofisher.com/antibody/product/Donkey-anti-Mouse-IgG-H-L-Secondary-Antibody-Polyclonal/R37114
Donkey anti-Mouse IgG (H+L) Highly Cross-Adsorbed Secondary Antibody, Alexa Fluor 555: https://www.thermofisher.com/antibody/product/Donkey-anti-Mouse-IgG-H-L-Highly-Cross-Adsorbed-Secondary-Antibody-Polyclonal/A-31570
Donkey anti-Goat IgG (H+L) Cross-Adsorbed Secondary Antibody, Alexa Fluor 555: https://www.thermofisher.com/antibody/product/Donkey-anti-Goat-IgG-H-L-Cross-Adsorbed-Secondary-Antibody-Polyclonal/A-21432
Donkey anti-Rabbit IgG (H+L) Highly Cross-Adsorbed Secondary Antibody, Alexa Fluor 488: https://www.thermofisher.com/antibody/product/Donkey-anti-Rabbit-IgG-H-L-Highly-Cross-Adsorbed-Secondary-Antibody-Polyclonal/A-21206
Donkey anti-Goat IgG (H+L) Cross-Adsorbed Secondary Antibody, Alexa Fluor 488: https://www.thermofisher.com/antibody/product/Donkey-anti-Goat-IgG-H-L-Cross-Adsorbed-Secondary-Antibody-Polyclonal/A-11055

Eukaryotic cell lines

Policy information about cell lines

Cell line source(s)	HEK293T cells were purchased from ATCC (CRL-3216); E14-NKX2-5-EmGFP ESCs generated by Hsiao et al as described in : Marking embryonic stem cells with a 2A self-cleaving peptide: a NKX2-5 emerald GFP BAC reporter. PLoS One 3, e2532 (2008), were provided by Prof. Thomas Braun (Max Planck Institute for Heart and Lung Research, Bad Nauheim)
Authentication	E14-NKX2-5-EmGFP ESCs line authentication was based on the detection of cellular green fluorescent protein. HEK293T cells were authenticated by ATCC.
Mycoplasma contamination	Cell lines were mycoplasma negative.
Commonly misidentified lines (See ICLAC register)	No commonly misidentified lines were used in the study.

Animals and other organisms

Policy information about studies involving animals; ARRIVE guidelines recommended for reporting animal research

Laboratory animals	The Lmna tm1.1Yxz/J line and the Gata4 tm1.1Sad were obtained from Jackson Laboratory and were maintained on a C57BL/6J background. Both male and female mice at the indicated in the figure legends age were used within the study. Mice were housed in a pathogen-free animal facility under standard conditions with a 12 hour light/dark cycle, temperature of 20-25 degrees and humidity range of 30-70%. All animal experiments were performed according to the regulations issued by the Committee for Animal Rights Protection of the State of Baden-Württemberg (Regierungspraesidium Karlsruhe, permit number: G-194/18).
Wild animals	No wild animals were used in this study.
Field-collected samples	No field-collected samples were used in this study.
Ethics oversight	All animal experiments were performed according to the regulations issued by the Committee for Animal Rights Protection of the State of Baden-Württemberg (Regierungspraesidium Karlsruhe, permit number: G-194/18).

Note that full information on the approval of the study protocol must also be provided in the manuscript.

ChIP-seq

Data deposition

☒ Confirm that both raw and final processed data have been deposited in a public database such as GEO.

☒ Confirm that you have deposited or provided access to graph files (e.g. BED files) for the called peaks.

Data access links<br>*May remain private before publication.*	GSE164069<br>https://www.ncbi.nlm.nih.gov/geo/query/acc.cgi?acc=GSE164069<br>password: ctkfwieyzjgpbwn
Files in database submission	GSM4995991 ESC_LMNA_ctr_RNA_seq_Rep1

Files in database submission

GSM4995992 ESC_LMNA_ctr_RNA_seq_Rep2
GSM4995993 ESC_LMNA_ctr_RNA_seq_Rep3
GSM4995994 ESC_LMNA_KO_RNA_seq_Rep1
GSM4995995 ESC_LMNA_KO_RNA_seq_Rep2
GSM4995996 ESC_LMNA_KO_RNA_seq_Rep3
GSM4995997 Day6_CPC_sorted_LMNA_ctr_RNA_seq_Rep1
GSM4995998 Day6_CPC_sorted_LMNA_ctr_RNA_seq_Rep2
GSM4995999 Day6_CPC_sorted_LMNA_ctr_RNA_seq_Rep3
GSM4996000 Day6_CPC_sorted_LMNA_KO_RNA_seq_Rep1
GSM4996001 Day6_CPC_sorted_LMNA_KO_RNA_seq_Rep2
GSM4996002 Day6_CPC_sorted_LMNA_KO_RNA_seq_Rep3
GSM4996003 Day10_EBs_LMNA_ctr_RNA_seq_Rep1
GSM4996004 Day10_EBs_LMNA_ctr_RNA_seq_Rep2
GSM4996005 Day10_EBs_LMNA_ctr_RNA_seq_Rep3
GSM4996006 Day10_EBs_LMNA_KO_RNA_seq_Rep1
GSM4996007 Day10_EBs_LMNA_KO_RNA_seq_Rep2
GSM4996008 Day10_EBs_LMNA_KO_RNA_seq_Rep3
GSM4996009 Day10_sorted_CM_LMNA_ctr_RNA_seq_Rep1
GSM4996010 Day10_sorted_CM_LMNA_ctr_RNA_seq_Rep2
GSM4996011 Day10_sorted_CM_LMNA_ctr_RNA_seq_Rep3
GSM4996012 Day10_sorted_CM_LMNA_KO_RNA_seq_Rep1
GSM4996013 Day10_sorted_CM_LMNA_KO_RNA_seq_Rep2
GSM4996014 Day10_sorted_CM_LMNA_KO_RNA_seq_Rep3
GSM4996015 Day10_CM_LMNA_KD_RNA_seq_Rep1
GSM4996016 Day10_CM_LMNA_KD_RNA_seq_Rep2
GSM4996017 Day10_CM_LMNA_KD_RNA_seq_Rep3
GSM4996018 Day10_CM_LMNA_PLKO_RNA_seq_Rep1
GSM4996019 Day10_CM_LMNA_PLKO_RNA_seq_Rep2
GSM4996020 Day10_CM_LMNA_PLKO_RNA_seq_Rep3
GSM4996021 ESC_LMNA_ctr_ATAC_seq_Rep1
GSM4996022 ESC_LMNA_ctr_ATAC_seq_Rep2
GSM4996023 ESC_LMNA_ctr_ATAC_seq_Rep3
GSM4996024 ESC_LMNA_KO_ATAC_seq_Rep1
GSM4996025 ESC_LMNA_KO_ATAC_seq_Rep2
GSM4996026 ESC_LMNA_KO_ATAC_seq_Rep3
GSM4996027 CM_LMNA_ctr_ATAC_seq_Rep1
GSM4996028 CM_LMNA_ctr_ATAC_seq_Rep2
GSM4996029 CM_LMNA_ctr_ATAC_seq_Rep3
GSM4996030 CM_LMNA_KO_ATAC_seq_Rep1
GSM4996031 CM_LMNA_KO_ATAC_seq_Rep2
GSM4996032 CM_LMNA_KO_ATAC_seq_Rep3
GSM4996033 ESC_LMNA_ctr_HIC_seq_Rep1
GSM4996034 ESC_LMNA_ctr_HIC_seq_Rep2
GSM4996035 ESC_LMNA_ctr_HIC_seq_Rep3
GSM4996036 ESC_LMNA_KO_HIC_seq_Rep1
GSM4996037 ESC_LMNA_KO_HIC_seq_Rep2
GSM4996038 ESC_LMNA_KO_HIC_seq_Rep3
GSM6438105 ESC_Lmna_ctr_fromMUT_ATAC_seq_Rep1
GSM6438106 ESC_Lmna_ctr_fromMUT_ATAC_seq_Rep2
GSM6438107 ESC_Lmna_G609G_het_ATAC_seq_Rep1
GSM6438108 ESC_Lmna_G609G_het_ATAC_seq_Rep2
GSM6438109 ESC_Lmna_G609G_hom_ATAC_seq_Rep1
GSM6438110 ESC_Lmna_H222P_hom_ATAC_seq_Rep1
GSM6438111 ESC_Lmna_H222P_hom_ATAC_seq_Rep2

Genome browser session
(e.g. UCSC)

To review GEO accession GSE164069:
Please, go to https://www.ncbi.nlm.nih.gov/geo/query/acc.cgi?acc=GSE164069
Enter token kjqdwwggbjqzxeb into the box.

Methodology

Replicates

RNA-Seq, ATAC-Seq, HiC experiments were performed with three independent biological replicates.

Sequencing depth

ESC_LMNA_KO_RNA_seq_Rep1 25030823
ESC_LMNA_KO_RNA_seq_Rep2 25036526
ESC_LMNA_KO_RNA_seq_Rep3 25032610
ESC_LMNA_ctr_RNA_seq_Rep1 25112914
ESC_LMNA_ctr_RNA_seq_Rep2 25038347
ESC_LMNA_ctr_RNA_seq_Rep3 25021622
Day6_CPC_sorted_LMNA_KO_RNA_seq_Rep1 27098613
Day6_CPC_sorted_LMNA_KO_RNA_seq_Rep2 26965337
Day6_CPC_sorted_LMNA_KO_RNA_seq_Rep3 26980409
Day6_CPC_sorted_LMNA_ctr_RNA_seq_Rep1 24710148
Day6_CPC_sorted_LMNA_ctr_RNA_seq_Rep2 26987286

Day6_CPC_sorted_LMNA_ctr_RNA_seq_Rep3 27090441
Day10_EBs_LMNA_KO_RNA_seq_Rep1 24395243
Day10_EBs_LMNA_KO_RNA_seq_Rep2 24486953
Day10_EBs_LMNA_KO_RNA_seq_Rep3 24574024
Day10_EBs_LMNA_ctr_RNA_seq_Rep1 24942892
Day10_EBs_LMNA_ctr_RNA_seq_Rep2 24662502
Day10_EBs_LMNA_ctr_RNA_seq_Rep3 24522606
Day10_sorted_CM_LMNA_KO_RNA_seq_Rep1 25014322
Day10_sorted_CM_LMNA_KO_RNA_seq_Rep2 25035647
Day10_sorted_CM_LMNA_KO_RNA_seq_Rep3 25066305
Day10_sorted_CM_LMNA_ctr_RNA_seq_Rep1 26203645
Day10_sorted_CM_LMNA_ctr_RNA_seq_Rep2 25069628
Day10_sorted_CM_LMNA_ctr_RNA_seq_Rep3 25063092
Day10_CM_LMNA_KD_RNA_seq_Rep1 25321512
Day10_CM_LMNA_KD_RNA_seq_Rep2 25945141
Day10_CM_LMNA_KD_RNA_seq_Rep3 24981534
Day10_CM_LMNA_PLKO_RNA_seq_Rep1 24855112
Day10_CM_LMNA_PLKO_RNA_seq_Rep2 24876409
Day10_CM_LMNA_PLKO_RNA_seq_Rep3 25389306
CM_LMNA_KO_ATAC_seq_Rep1_R1 29049608
CM_LMNA_KO_ATAC_seq_Rep1_R2 29049608
CM_LMNA_KO_ATAC_seq_Rep2_R1 24059618
CM_LMNA_KO_ATAC_seq_Rep2_R2 24059618
CM_LMNA_KO_ATAC_seq_Rep3_R1 26123700
CM_LMNA_KO_ATAC_seq_Rep3_R2 26123700
CM_LMNA_ctr_ATAC_seq_Rep1_R1 24057159
CM_LMNA_ctr_ATAC_seq_Rep1_R2 24057159
CM_LMNA_ctr_ATAC_seq_Rep2_R1 27228506
CM_LMNA_ctr_ATAC_seq_Rep2_R2 27228506
CM_LMNA_ctr_ATAC_seq_Rep3_R1 27291437
CM_LMNA_ctr_ATAC_seq_Rep3_R2 27291437
ESC_LMNA_KO_HIC_seq_Rep1_R2 29512449
ESC_LMNA_KO_HIC_seq_Rep2_R1 70336750
ESC_LMNA_KO_HIC_seq_Rep2_R2 70336750
ESC_LMNA_KO_HIC_seq_Rep3_R1 57839753
ESC_LMNA_KO_HIC_seq_Rep3_R2 57839753
ESC_LMNA_ctr_HIC_seq_Rep1_R1 59807247
ESC_LMNA_ctr_HIC_seq_Rep1_R2 59807247
ESC_LMNA_ctr_HIC_seq_Rep2_R1 75151396
ESC_LMNA_ctr_HIC_seq_Rep2_R2 75151396
ESC_LMNA_ctr_HIC_seq_Rep3_R1 42738266
ESC_LMNA_ctr_HIC_seq_Rep3_R2 42738266

Antibodies

Antibodies were not used in RNA-seq, ATAC-seq and HiC-seq

Peak calling parameters

For ATAC-seq
MACS2 (-q 0.01 --nomodel --shift -75 --extsize150)

Data quality

FastQC

Software

STAR (v2.7.1a), DeepTools(v3.3.0), MACS(v2.1.1),

Flow Cytometry

Plots

Confirm that:

☒ The axis labels state the marker and fluorochrome used (e.g. CD4-FITC).

☒ The axis scales are clearly visible. Include numbers along axes only for bottom left plot of group (a 'group' is an analysis of identical markers).

☒ All plots are contour plots with outliers or pseudocolor plots.

☒ A numerical value for number of cells or percentage (with statistics) is provided.

Methodology

Sample preparation

For FACS staining of extracellular markers, EBs were washed with HBSS twice and dissociated into single cells by incubation with 1 mg/ml collagenase I (Cell Systems, LS004196) at 37°C for 30 min. Cells were then washed with 5% FCS/PBS and blocked in 10% FCS/PBS buffer for 30 min at room temperature. 200 000 cells were used for staining with 2.5 µl APC-conjugated anti-Flk1 (e-Bioscience, 17-5821-81,1:40), PE-conjugated anti-PDGFRα (e-Bioscience, 12-1401-81,1:40), APC-

conjugated anti-Pecam1 (Thermo Fisher Scientific, 17-0311-82,1:40) antibody in 100 µl FACS buffer (0.4% BSA) for 1 hour at room temperature in a dark place. After washing with FACS buffer twice, cells were resuspended in 300 µl FACS buffer and subjected to analysis by BD FACSDiva Software (version8.0.1, firmware version 1.49 BD FACSCanto II).
For FACS staining of intracellular cardiac Troponin T, single cells were prepared as above. After washing with 5% FCS/PBS, 400 000 cells were fixed in 500 µl 3.7% PFA for 30 min at RT. Cells were then washed with 5% FCS/PBS buffer and incubated in permeabilization buffer (0.5% saponin/5% FCS/PBS) for 15 minutes on ice. Cells were further stained with 2.5 µl APC-conjugated anti-troponin T antibody (BD, 1:40) in 100 µl permeabilization buffer for 2 hours at RT in a dark place, followed by washes with permeabilization buffer, PBS and FACS buffer and resuspended in 300 µl FACS buffer for FACS analysis.
For apoptosis assay by FACS, one million cells were used for staining with 5 µl of APC Annexin V and 5 µl of 7-AAD in 100 µl Annexin V binding buffer for 15 min at room temperature according to the APC Annexin V Apoptosis Detection Kit with 7-AAD (Biolegend, 640930) instructions. Cells were then resuspended in 400 µl of Annexin V Binding Buffer and subjected to FACS analysis. CMs and non-CMs were distinguished by Nkx2-5 GFP expression.
To assess CM binucleation by FACS, E14.5 and E18.5 hearts were freshly harvested, washed with cold HBSS and dissociated into single cells by incubation with digestion buffer (1 mg/ml collagenase I and 1mg/ml DNase I in HBSS) at 37°C 30 min. Cell suspension was then applied to a 70µm cell strainer to remove cell debris. Single cells were stained with APC-conjugated anti-Pecam1 antibody for 30 min at 4 °C followed by washing with cold HBSS/10%FCS three times. Cells were then stained with Vybrant™ DyeCycle™ Green (Thermo Fisher Scientific, 1:500 dilutions by HBSS/10%FCS) at 37 °C for 30 min and were subjected to FACS analysis.

Instrument

BD FACSCanto II, BD FACSAria IIu

Software

Data were collected and analyzed using BD FACSDivaTM software(version8.0.1).

Cell population abundance

Post sorting confirmed the high purity of the sorted populations.

Gating strategy

Unstained cells were used to define negative cell populations and set gates for analysis.

☒ Tick this box to confirm that a figure exemplifying the gating strategy is provided in the Supplementary Information.
